# Artificial Intelligence in Pancreatic Resection: Current Evidence, Surgical Applications, and a Framework for Clinical Translation

**DOI:** 10.3390/jcm15218737

**Published:** 2026-11-09

**Authors:** Karim Heiba, Ambareen Kausar

**Affiliations:** Department of General Surgery, Royal Blackburn Teaching Hospital, East Lancashire Hospitals NHS Trust, Haslingden Road, Blackburn BB2 3HH, UK; karim.heiba@elht.nhs.uk

**Keywords:** artificial intelligence, deep learning, machine learning, pancreatic resection, pancreatoduodenectomy, distal pancreatectomy, postoperative pancreatic fistula, radiomics, three-dimensional reconstruction, robotic surgery

## Abstract

**Introduction:** Pancreatic resection remains technically demanding, with clinically relevant postoperative pancreatic fistula (CR-POPF) a major source of morbidity. Artificial intelligence (AI) may support risk stratification, operative planning, intraoperative assessment, and postoperative decision support. This focused narrative review evaluates current AI applications in pancreatic resection and their translational maturity. **Methods:** A structured PubMed/MEDLINE search with an evidence cutoff of 24 July 2026 was supplemented by backward and forward citation tracking. Seventy eligible primary reports were synthesised by clinical task, information timing, and validation maturity. **Results:** Evidence is dominated by CR-POPF prediction after pancreatoduodenectomy, with additional studies in distal pancreatectomy, other perioperative outcomes, tissue and margin assessment, three-dimensional anatomical planning, and intraoperative video and anatomy recognition. Performance varied with patient selection, predictor timing, data modality, and validation strategy. External validation often changed reported discrimination, while calibration and clinical utility were inconsistently reported. Most studies were retrospective or technical; overlapping cohorts limited independence, and no included study demonstrated improved patient-centred outcomes. **Conclusions:** AI in pancreatic resection is expanding across prediction, planning, and guidance, but technical performance should not be equated with clinical readiness. Independent validation, prospective workflow and human-factors evaluation, and clinical-impact studies are needed. AI should augment, rather than replace, specialist surgical judgement.

## 1. Introduction

Pancreatic resections are technically demanding and require coordinated decisions before, during, and after surgery, but are still associated with substantial postoperative morbidity. Artificial intelligence (AI) has emerged as a potential means of integrating complex clinical, imaging, pathological, and procedural data to support prediction, planning, and perioperative decision-making in this setting.

Among the major complications of pancreatic resection, clinically relevant postoperative pancreatic fistula (CR-POPF), defined by the 2016 International Study Group of Pancreatic Surgery criteria, is associated with sepsis, haemorrhage, prolonged recovery, reintervention, and death [1]. Existing fistula-risk scores are valuable but have limitations. The Fistula Risk Score and the alternative Fistula Risk Score combine clinical and operative variables, yet their performance varies across institutions, and external validation studies have reported only modest discrimination [2,3,4]. These findings highlight the challenges of predicting outcomes using conventional approaches alone. In pancreatic surgery, machine-learning and deep-learning methods have been applied to CR-POPF prediction, body-composition analysis, pancreatic and vascular segmentation, three-dimensional reconstruction, and surgical video assessment. Such approaches may capture features that are difficult to quantify reproducibly using routine assessment.

Previous reviews have examined AI across pancreatic disease broadly, including diagnosis, staging, and surveillance [5,6]. The literature relevant to pancreatic resection has since expanded across several distinct tasks: clinical prediction and decision support, surgical-margin and tissue assessment, anatomical segmentation and three-dimensional (3D) planning, and intraoperative computer-vision or workflow analysis. This review therefore focuses specifically on pancreatic resection, separates these technologies according to their clinical task and predictor timing, and emphasises the evidentiary steps required before technical performance can be translated into clinical use.

We use prediction, planning, and guidance as a clinical organising framework. Translational maturity is considered separately, according to progression from development and internal validation to independent external validation, prospective workflow and human-factors evaluation, and clinical-impact assessment. The framework is not proposed as a new regulatory pathway or a validated implementation algorithm.

## 2. Methods

A structured PubMed/MEDLINE search was conducted to identify studies evaluating artificial-intelligence (AI)-based approaches in the perioperative and technical aspects of pancreatic resection. A predefined evidence cutoff of 24 July 2026 was applied using a PubMed Create Date restriction. The complete final Boolean expression, including all Title/Abstract field tags, Boolean operators, and the date restriction, is reproduced in full below Table 1. Reference lists of eligible primary studies and relevant reviews were screened, and backward and forward citation tracking was used to identify additional peer-reviewed reports.

Eligible primary reports were English-language studies evaluating AI, machine learning, deep learning, radiomics, computer vision, AI-assisted 3D methods, or computational tissue-classification approaches with a direct perioperative or technical application to pancreatic resection. Eligible applications included complication prediction, operative planning, intraoperative guidance or recognition, margin assessment, and early postoperative decision support.

Studies focused exclusively on diagnosis, pancreatic cyst classification, long-term oncological outcomes, recurrence prediction, long-term metabolic sequelae, or nodal staging without a direct perioperative or technical endpoint were excluded, as were non-pancreatic or non-separable mixed cohorts, conventional statistical analyses without an eligible computational method, animal studies, conference abstracts, non-peer-reviewed preprints, non-primary reports, and non-English publications. Where overlapping cohorts were identified, studies were retained only when they reported distinct outcomes, validation cohorts, modalities, algorithms, or clinical applications.

The search identified 1613 unique records. No article-type or human-species filters were applied at the search stage. Following title and abstract screening, 220 reports underwent full-text assessment for eligibility. After application of the predefined eligibility criteria, 68 studies were included from the database search. Backward and forward citation tracking identified two additional eligible studies not retrieved by the final search strategy, resulting in a final qualitative synthesis of 70 reports. The study-selection process is summarised in Supplementary Figure S1, with reasons for full-text exclusion reported in Supplementary Table S2.

Data were extracted from eligible studies and summarised descriptively. Owing to substantial heterogeneity in procedures, outcomes, input modalities, algorithms, validation approaches, and performance reporting, findings were synthesised narratively, and no quantitative meta-analysis was performed.

Given the heterogeneity of the evidence base, spanning prediction modelling, radiomics, segmentation, computer vision, 3D modelling, and computational tissue assessment, no single risk-of-bias instrument was applied. Instead, appraisal was informed by TRIPOD + AI, PROBAST + AI, and DECIDE-AI principles, with attention to patient selection, predictor timing, sample size and event rates, overfitting, data leakage, calibration, external validation, threshold-dependent performance, decision-curve analysis, and evidence of clinical utility [7,8,9]. Studies were further categorised as development/internal validation, external or temporal validation, prospective evaluation, or technical/feasibility evidence, with classifications reported in Supplementary Table S1.

Full text was used where available and required for interpretation; when full text was inaccessible, only explicitly indexed information was used, and unreported details were not inferred.

## 3. Concepts and Appraisal of AI for Surgeons

### 3.1. Artificial Intelligence, Machine Learning, and Deep Learning

Artificial intelligence (AI) refers to computational systems that perform tasks such as prediction, classification, image interpretation, and decision support. Machine learning (ML) is a subset of AI that learns patterns from data rather than relying solely on predefined rules. Most pancreatic-surgery studies use supervised learning, in which models are trained to predict outcomes such as clinically relevant postoperative pancreatic fistula (CR-POPF).

Deep learning (DL) is a machine-learning approach that uses multilayer neural networks to learn patterns directly from complex data. It is particularly well suited to high-dimensional inputs such as CT imaging and surgical video. However, clinical utility depends less on model complexity than on accuracy, calibration, interpretability, and validation in the target population.

### 3.2. Radiomics and Imaging-Derived Features

Radiomics converts medical images into quantitative descriptors of shape, intensity, texture, and spatial organisation [10]. These features can be analysed using conventional statistical or machine-learning methods to characterise pancreatic parenchyma, ductal anatomy, remnant morphology, and surrounding tissues. Because radiomic analyses are sensitive to imaging acquisition and segmentation methods, standardisation is essential for reproducibility [11].

### 3.3. Computer Vision and Three-Dimensional Modelling

Computer vision enables automated interpretation of images and video, including recognition of anatomy, instruments, and operative phases. Three-dimensional (3D) modelling uses imaging data to reconstruct pancreatic, ductal, tumour, and vascular anatomy for measurement and surgical planning. These image-processing tasks should be distinguished from clinical prediction models: segmentation accuracy or phase-recognition accuracy demonstrates technical performance, while prediction models estimate a patient-level outcome probability. Technical accuracy alone does not establish clinical value; evidence of improved planning, decision-making, or patient outcomes is required [12].

### 3.4. Clinical Task, Information Timing, and Terminology

For this review, AI applications are separated by clinical task rather than grouped solely by algorithm: patient-level prediction or decision support; radiomics and other imaging-derived prediction; anatomical segmentation and AI-assisted three-dimensional planning; intraoperative computer vision, anatomy recognition, or workflow analysis; and computational pathology or tissue and margin assessment. These categories may use similar machine-learning methods, but their outputs, validation requirements, and routes to clinical use differ.

For prediction models, the information timepoint is defined by the latest variable required to generate the output. A model is preoperative only when all required inputs are available before incision; models requiring gland texture, blood loss, fluid administration, or operative findings are intraoperative or mixed-timepoint, while those using drain amylase or postoperative trajectory are early postoperative. Histological features are post-resection information unless a validated preoperative surrogate exists. Offline segmentation, phase recognition, anatomical labelling, and retrospective 3D visualisation are therefore described as analysis, recognition, or planning tools; ‘guidance’ is reserved for systems that deliver information at a clinically relevant decision point and are evaluated for their effect on decisions, workflow, safety, or outcomes.

### 3.5. Framework for Interpreting Model Performance

In this review, prediction-model performance was interpreted across discrimination, calibration, threshold performance, external validation, and clinical utility rather than by the area under the receiver-operating-characteristic curve (AUC) alone. Discrimination, commonly reported using the AUC, was considered as a measure of how well a model ranks patients with and without an outcome, but not as evidence that predicted probabilities are numerically accurate. Where reported, calibration measures, sensitivity and specificity, positive and negative predictive values, precision–recall measures, and decision-curve analysis were therefore considered alongside discrimination. Sample size, number of outcome events, and model complexity were also considered when interpreting the risk of optimism and overfitting [7,8,9].

For CR-POPF models, reported threshold performance was interpreted in relation to the clinical decision the model was intended to support. False-negative predictions were considered particularly relevant where a low-risk classification might lead to reduced surveillance, earlier drain removal, or deferred investigation, while false-positive predictions could result in unnecessary drainage, imaging, monitoring, or delayed discharge. Decision-curve analysis, when available, was interpreted as evidence of potential net benefit at specified thresholds rather than as proof that model-guided management improves clinical outcomes.

External or temporal validation was treated as stronger evidence of transportability than internal validation alone. Changes in performance between development and validation cohorts were interpreted in the context of differences in CR-POPF prevalence, pathology, operative practice, imaging acquisition, variable definitions, and case mix. Attention was given to whether discrimination was maintained, whether calibration was reported, and whether any clinical-impact evaluation had been undertaken. These considerations informed the maturity classifications used throughout the evidence synthesis and in Supplementary Table S1.

## 4. Current Evidence in Pancreatic Resection

The final principal synthesis comprised 70 primary reports. By primary task, 34 addressed POPF prediction or fistula-related decision support, 14 addressed other perioperative prediction or guidance, eight addressed surgical-margin or tissue assessment, eight addressed intraoperative computer vision or workflow recognition, and six addressed anatomical segmentation or 3D planning. These counts describe reports rather than independent cohorts because registry and institutional overlap is present. The evidence is therefore discussed by clinical task, predictor timing, and validation maturity rather than with all methods treated as equivalent. Selected representative and higher-maturity POPF studies are summarised in Table 2, which is intentionally representative rather than exhaustive: studies were selected for external or temporal validation, prospective evaluation, direct comparison with established tools, or representation of a clinically distinct modality.

All eligible reports are listed in Supplementary Table S1. Information timepoint refers to the latest variable required to generate the reported prediction; a mixed-timepoint model is not considered a substitute for a purely preoperative model. Missing-data handling, class-imbalance strategies, and related development details were inconsistently reported across source studies and were not inferred when unavailable.

### 4.1. POPF Prediction After Pancreatoduodenectomy

Early CT and MRI radiomics studies showed that routine imaging contains quantitative information associated with postoperative pancreatic fistula, although the highest reported AUCs came from selected single-centre cohorts [13,23,24]. Mu et al. subsequently developed a contrast-enhanced CT deep-learning score in 513 patients from three institutions and prospectively evaluated it in 70 patients at a fourth centre; the external-test AUC was 0.89 compared with 0.73 for the conventional Fistula Risk Score (FRS) [14]. The score was also associated with recognised pancreatic characteristics, including fibrosis and duct size, supporting biological interpretability. This remains an important demonstration that a genuinely preoperative imaging model can capture clinically relevant pancreatic characteristics, but the small external cohort limits certainty about transportability.

Clinical-variable models span different information timepoints and should not be treated as interchangeable. Han et al. reported a maximal AUC of 0.74 in 1769 pancreatoduodenectomies, but the platform incorporated intraoperative variables, including gland texture and fluid administration, and is therefore a mixed preoperative–intraoperative tool [25]. External validation in 1576 patients from three centres produced AUCs of 0.585–0.672 [15]. By contrast, Ganjouei et al. and Matsui et al. evaluated models using only preoperatively available variables [17,26]. Verma et al. trained XGBoost in 12,281 ACS-NSQIP cases and achieved an external AUC of 0.79 (95% CI 0.74–0.84) in 445 institutional patients versus 0.75 for the modified FRS; internal AUPRC was 0.22 versus 0.18 [18].

Radiomic–clinical and multimodal approaches have also shown encouraging but heterogeneous results. Capretti et al. and Bhasker et al. reported internally validated AUCs of 0.807 and 0.86 in small single-centre cohorts [27,28]. The externally validated RAD-FRS achieved an AUC of 0.81 (95% CI 0.69–0.92), with sensitivity 0.96, specificity 0.66, PPV 0.64, NPV 0.96, and acceptable calibration; discrimination was similar to that of the FRS and updated a-FRS (0.79 for each) [16]. Lee et al. reported an AUC of 0.68 for CR-POPF in temporal testing [19]. Other contemporary models using radiomics, body composition, MRI, or automated feature extraction have reported promising internal or selected prospective performance, but most still lack independent external confirmation [20,29,30,31,32,33].

Comparison with established clinical scores is therefore central. The original FRS is simple and interpretable but depends on intraoperative gland texture, duct diameter, pathology, and blood loss; the a-FRS is simpler but still requires intraoperative assessment of gland texture [2,3]. A meta-analysis of external validations reported pooled AUROCs of approximately 0.71 for the FRS, 0.70 for the a-FRS, and 0.72 for the updated a-FRS [4]. AI has not consistently exceeded these benchmarks: Mu et al. reported a substantial advantage over the FRS in a selected external cohort [14], whereas RAD-FRS and AutoFRS were similar to conventional comparators [16,20], nationwide structured-data modelling showed little difference between machine learning and logistic regression [34], and Verma et al. demonstrated only a modest advantage over the modified FRS [18]. The clearest potential advantage of AI may therefore be objective, automated, genuinely preoperative multimodal assessment rather than universally superior discrimination; this potential must be weighed against greater imaging, software, integration, and validation requirements than those of simple bedside scores.

The external-validation studies illustrate variable transportability across settings. The AI-based POPF risk-prediction platform developed by Han et al. at Samsung Medical Center fell to AUCs of 0.585–0.672 across three external centres [15]; RAD-FRS decreased from 0.90 internally to 0.81 externally while retaining acceptable calibration [16]; AutoFRS changed from 0.81 to 0.79 [20]; and the Verma model performed better in its external institutional cohort than in internal validation [18]. These contrasting directions may reflect differences in case mix, outcome prevalence, imaging, operative practice, and variable definitions, and emphasise that external performance cannot be inferred reliably from development results alone.

Across the CR-POPF literature, discrimination is reported more consistently than calibration, threshold performance, predictive values, or decision-curve analysis. Some models have shown statistical net benefit on decision-curve analysis [21,29], while predictor timing varies from purely preoperative to mixed perioperative inputs. This distinction is clinically important because the same model output may support different decisions depending on when its predictors become available. To date, included studies have primarily evaluated predictive performance rather than the effect of AI-informed management on patient-centred outcomes.

### 4.2. Prediction After Distal Pancreatectomy

Distal pancreatectomy presents distinct challenges because leakage originates from the transected pancreatic stump rather than a pancreaticoenteric anastomosis, and the AI evidence base is substantially smaller than that for pancreatoduodenectomy. Liang et al. studied 230 patients undergoing radical distal pancreatectomy for pancreatic cancer across two institutions; grade B/C POPF occurred in 47.4%. Their integrated preoperative clinical and body-composition model achieved an external-test AUC of 0.82, sensitivity of 0.81, and specificity of 0.76, with decision-curve analysis used to assess net benefit [21]. However, the external test set comprised only 42 patients, the cohort was restricted to pancreatic cancer, and tumour size and stapler use differed between the development and external cohorts. These features limit immediate generalisability despite encouraging external-test performance.

Miyamoto et al. evaluated 109 patients with pancreatic cancer at a single centre and found associations between AI-derived pancreatic-stump roundness, remnant-volume ratio, and POPF [22]. The morphometric measurements themselves are available from preoperative three-dimensional imaging, but the multivariable analysis also incorporated pathological fibrosis, which is only known after resection, and cases with inadequate segmentation were excluded. The study should therefore be interpreted as a mixed-timepoint association analysis that identifies potentially informative morphology rather than as a deployable preoperative probability model. External probability-model validation, calibration, and decision-curve analysis were not reported.

Taken together, the distal-pancreatectomy literature is smaller than the pancreatoduodenectomy evidence base but suggests that preoperative body composition and pancreatic morphology may provide useful predictive information. Current studies are modest in size, often restricted to selected cancer populations, and include event rates that may not transport directly to other services. Independent multicentre validation in broader distal-pancreatectomy populations and prospective evaluation linked to defined clinical decisions have not yet been reported.

### 4.3. Digital Pathology, Tissue Characterisation, and Surgical-Margin Assessment

Machine-learning analysis of pancreatic resection specimens can provide objective measures of gland composition. In a RECOPANC substudy, quantitative fibrosis outperformed subjective gland-texture assessment for CR-POPF prediction [35]. Because histological information is available only after transection, however, such measurements support post-resection risk assessment and biological characterisation rather than genuinely preoperative prediction.

Margin assessment is a distinct application. Preoperative CT radiomics and delta-radiomics have been used to estimate R0/R1 or vascular-margin status, including studies with external or prospective evaluation [36,37,38,39,40], while intraoperative computational tissue assessment has been explored using mass-spectrometry imaging and Raman-based molecular cytology [41,42]. These studies broaden the potential role of AI beyond fistula prediction and include preoperative, intraoperative, and pathology-based approaches. Current evidence primarily establishes predictive, diagnostic, or technical performance; direct evaluation of whether AI-assisted margin assessment improves resection quality or patient outcomes remains limited.

### 4.4. AI-Assisted Three-Dimensional Surgical Planning from Preoperative Imaging

**Figure 1:**
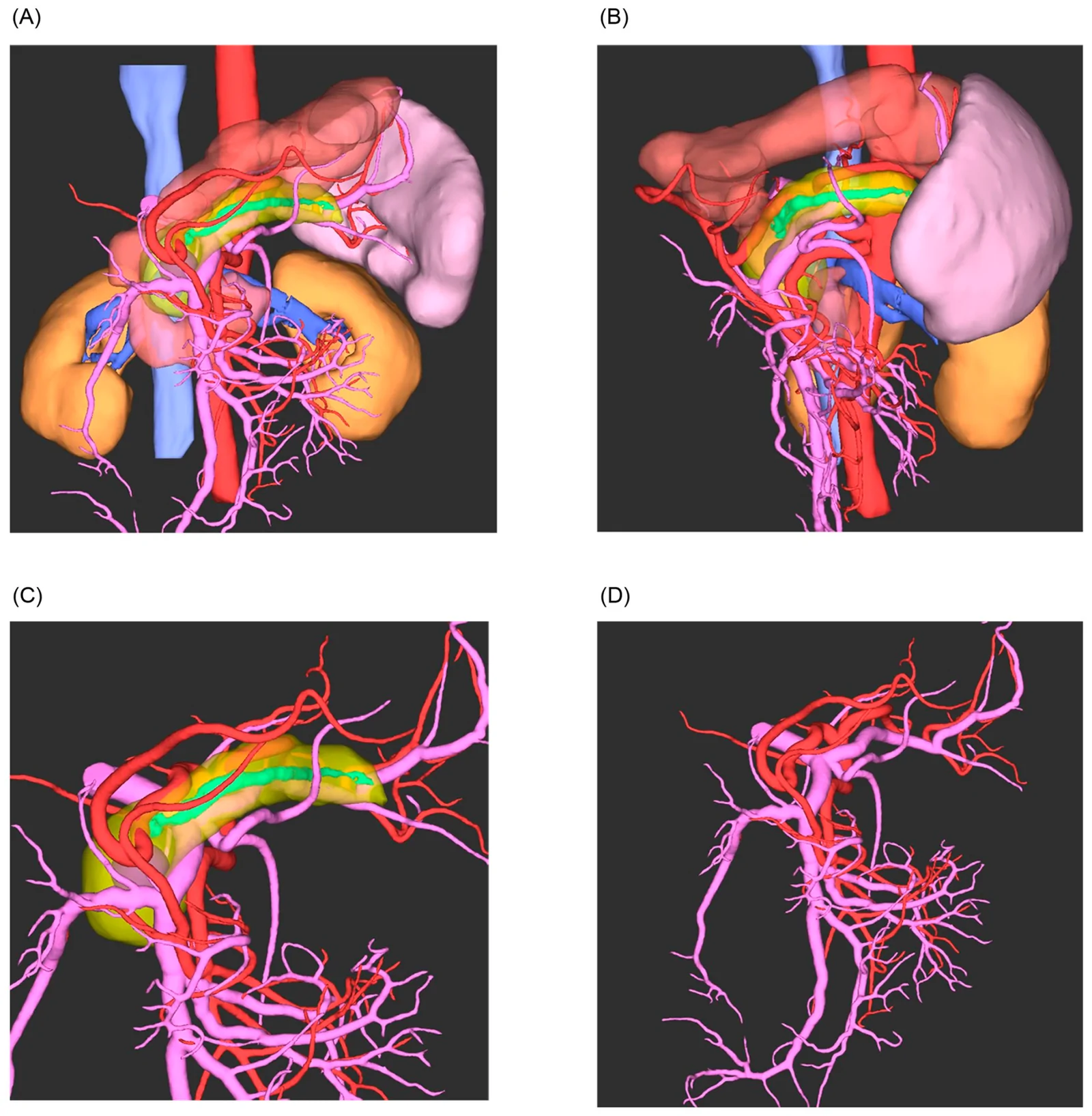
Artificial-intelligence (AI)-assisted three-dimensional simulation of pancreatic and peripancreatic anatomy. Panels (**A**,**B**) show anterior and left-lateral views. Panel (**C**) shows the pancreas, pancreatic duct, tumour, and peripancreatic arteries and veins; panel (**D**) isolates the peripancreatic vasculature. Red denotes arteries; pink, veins including the portal vein; yellow, pancreas; turquoise, pancreatic duct; blue, renal vein and inferior vena cava; dark yellow, kidney; light pink, spleen; copper, stomach and duodenum; and dark pink, pancreatic tumour. Reproduced without modification from Miyamoto et al. [43] under the Creative Commons Attribution 4.0 licence.

Deep-learning segmentation can reconstruct pancreatic, ductal, tumour, and vascular anatomy from preoperative imaging. Miyamoto et al. demonstrated technically strong pancreas and pancreatic-duct segmentation in 100 pancreatic-cancer patients and 100 controls [43], and multicentre work has subsequently shown external validation of automated 3D segmentation [44]. Related studies have used 3D models to quantify remnant morphology, vascular anatomy, and anticipated operative difficulty [22,45,46]. A single-case 3D computer-graphics report illustrates potential use for operative preparation but does not establish clinical navigation or benefit [47]. An example of AI-assisted three-dimensional anatomical reconstruction is shown in Figure 1.

Postoperative residual-pancreas segmentation has also been investigated [48], but because it uses postoperative imaging, it addresses a different use case from preoperative planning. Overall, current studies demonstrate the feasibility of automated segmentation and patient-specific 3D anatomical modelling. Evidence that these outputs alter operative planning, intraoperative decisions, complications, or patient outcomes is currently limited, so the technology is best described as AI-assisted planning or anatomical modelling rather than established real-time navigation.

### 4.5. Robotic Surgical Video and Anatomy Analysis

Surgical video analysis is an emerging application of AI in pancreatic resection. Al Abbas et al. analysed 100 robotic pancreaticojejunostomy videos and trained a three-dimensional convolutional neural network to recognise six procedural tasks, achieving 88.01% accuracy and a mean per-class F1 score of 85.34% [49]. Subsequent studies have extended this concept beyond pancreaticojejunostomy to laparoscopic phase recognition, major-vessel segmentation, intraoperative-ultrasound pancreatic-duct detection, and quantitative assessment of robotic superior mesenteric artery dissection [50,51,52,53,54]. Together, these studies show that complex pancreatic procedures can be analysed computationally at the level of workflow and anatomy.

Current studies support applications such as automated video indexing, structured operative review, training, quality improvement, and anatomy-aware analysis. The published work primarily evaluates phase recognition, frame-level anatomy detection, offline segmentation, or postoperative visualisation. Real-time guidance at a clinically relevant decision point is a distinct use case and has not yet been evaluated to the same extent in pancreatic surgery [49,50,51,52,53,54].

### 4.6. Postoperative Prediction and Decision Support

Early postoperative AI models incorporate dynamic information that is unavailable before surgery and therefore address a different decision point from preoperative risk stratification. Shen et al. reported an AUC of approximately 0.83 for CR-POPF prediction using serial postoperative variables, with drain-fluid amylase emerging as the strongest predictor [55]. Other models have been developed specifically to identify lower-risk patients during the early postoperative course for dynamic management [56].

Such models may support postoperative surveillance, drain management, imaging decisions, and escalation or de-escalation of care. They address a different decision point from genuinely preoperative risk models because their inputs include evolving postoperative information. Current studies mainly evaluate predictive performance; prospective pathway studies linking model outputs to specific postoperative management decisions remain limited.

### 4.7. Beyond POPF: Other Perioperative Outcomes

The evidence base also includes AI applications beyond fistula prediction, including wearable-data prediction of textbook outcome [57], structured-data models for multiple postoperative complications [34], early machine-learning-supported haemodynamic management [58], and models for post-discharge venous thromboembolism, post-pancreatoduodenectomy haemorrhage, acute pancreatitis, and mortality [59,60,61,62,63]. These studies broaden the potential perioperative role of AI and span development, external validation, decision-curve analysis, and early clinical evaluation, but they are heterogeneous in outcome and data source. Clinical-impact evidence remains sparse, so comparisons across these diverse endpoints should be made cautiously.

## 5. Clinical Applications Across the Patient Pathway

**Figure 2:**
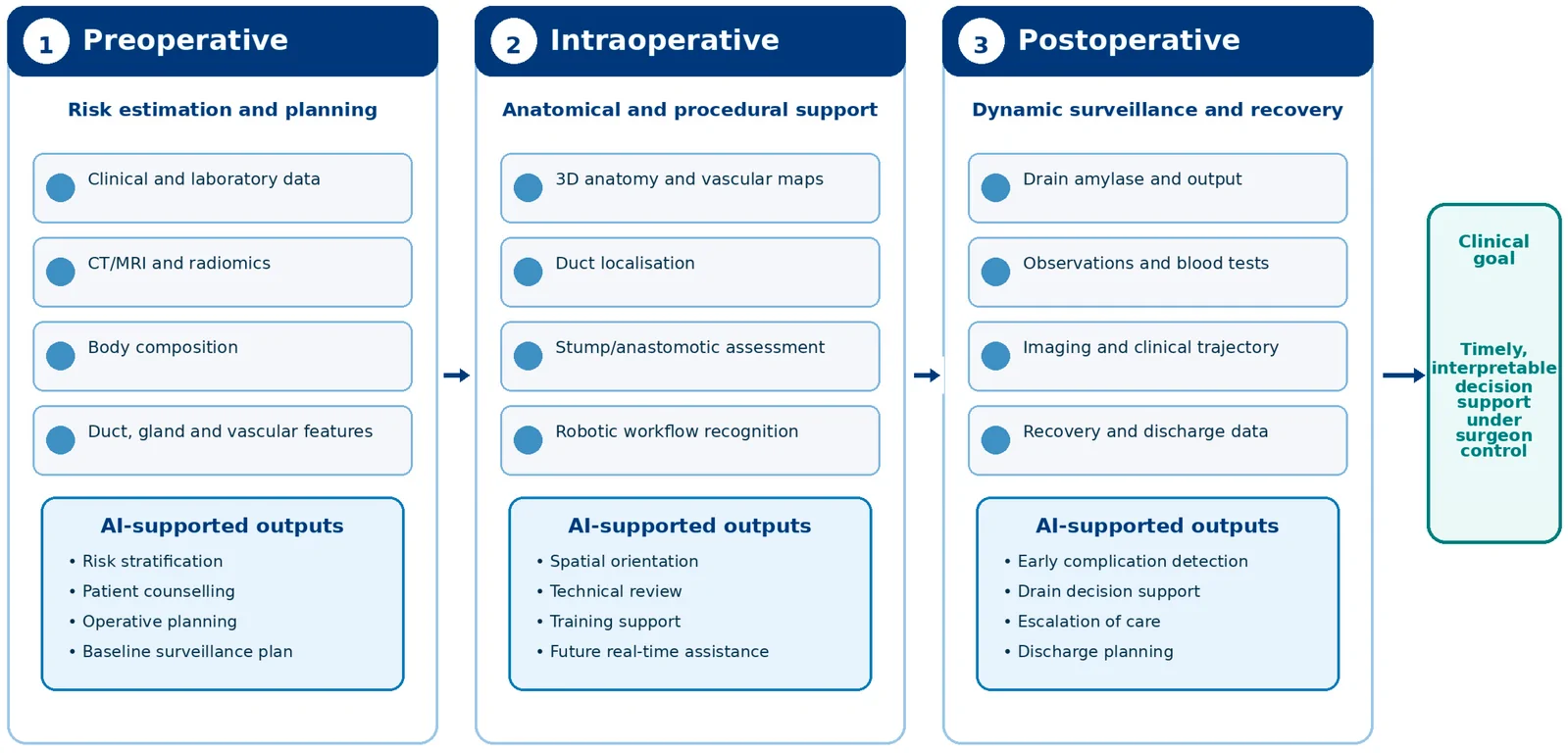
Artificial intelligence (AI) across the pancreatic-resection pathway. The diagram distinguishes data inputs, AI-supported outputs, and the intended clinical goal across preoperative, intraoperative, and postoperative phases. Most current evidence remains retrospective model development rather than implemented care. Original figure.

Current AI applications in pancreatic resection should be considered along two axes: the clinical task being performed and the timepoint at which the required information becomes available. Preoperative prediction and anatomical planning, intraoperative recognition or analysis, and early postoperative decision support can all use AI, but they support different decisions and require different validation. Figure 2 summarises the pathway, and Table 3 separates demonstrated technical capability from proposed clinical use. Real-time decision support and autonomous recommendations remain investigational.

### 5.1. Preoperative Phase

The most realistic near-term application is the generation of patient-specific risk and anatomy reports from routine clinical and imaging data. Such reports may combine fistula-risk estimates with quantitative measures of duct diameter, body composition, remnant morphology, vascular anatomy, margin-risk features, and anticipated operative difficulty to support counselling and multidisciplinary planning [39,40,44,45,46]. Potential risk-adapted strategies, including enhanced prehabilitation or intensified surveillance, remain hypotheses for prospective evaluation rather than established AI-informed pathways.

### 5.2. Intraoperative Phase

Three-dimensional reconstructions may improve understanding of patient-specific anatomy and support operative planning, particularly in minimally invasive surgery. Surgical video, vessel-recognition, and intraoperative-ultrasound models can currently support automated indexing, technical review, and experimental anatomy recognition [49,52,53,54]. However, real-time navigation and intraoperative alerts face additional challenges related to anatomical deformation, registration accuracy, false-positive detections, latency, workflow integration, and human factors.

### 5.3. Postoperative Phase

Postoperative models can integrate serial clinical variables, laboratory results, imaging findings, and drain-fluid measurements to estimate evolving fistula risk. Potential applications include drain management, escalation of care, and imaging decisions. The acceptable error profile depends on the intended decision, and prospective evaluation is required before incorporation into clinical pathways.

### 5.4. Human and Institutional Context

Pancreatic-surgery outcomes are strongly influenced by surgeon experience, institutional volume, rescue capability, and multidisciplinary infrastructure; a 2023 systematic review and meta-analysis confirmed an association between higher hospital and surgeon volume and lower postoperative mortality [64]. This matters for AI because models may encode local case selection, operative technique, imaging practice, drain management, and thresholds for intervention. Performance in a high-volume HPB centre should therefore not be assumed to transport unchanged to a differently organised service. External and prospective studies should report surgeon and centre context and assess calibration, usability, and decision impact across settings. AI should augment specialist judgement and team workflow rather than substitute for expertise [12,64].

## 6. Limitations

The current evidence base remains limited by methodological weaknesses. Most studies were retrospective, single-centre analyses, often based on relatively small datasets, creating substantial risks of selection bias, overfitting, and inflated performance estimates that may not generalise to independent populations. Several reports also used overlapping registries or institutional cohorts, meaning that the 70 included studies do not represent 70 independent datasets.

Considerable heterogeneity in outcomes, predictors, imaging protocols, algorithms, and validation methods further limits comparability between studies. Performance in one centre, dataset, or technical domain should therefore not be interpreted as evidence of broader clinical readiness. In addition, many models incorporate intraoperative or postoperative variables, making comparisons with genuinely preoperative prediction tools difficult.

A recurring weakness of the literature is its focus on discrimination metrics, particularly AUROC, with less consistent reporting of calibration, threshold performance, predictive values, and clinical utility. Consequently, technical performance frequently exceeds evidence of real-world usefulness. Notably, no included study demonstrated that AI-guided management reduces clinically relevant postoperative pancreatic fistula, overall complications, length of stay, or readmission, or improves survival. The field therefore remains dominated by development and validation studies rather than evidence of clinical impact.

This review also has limitations. Restriction to PubMed/MEDLINE and English-language peer-reviewed publications may have excluded relevant studies, particularly from the engineering and computer-science literature. Although citation tracking was performed to reduce this risk, some evidence may have been missed. Finally, the rapidly evolving nature of AI means that new models and validation studies may have emerged after the review cutoff date, and the heterogeneity of the available evidence precluded quantitative synthesis.

## 7. Future Directions and Translational Readiness

### 7.1. Staged Evaluation Pathway

Translation of AI into pancreatic surgery should follow a staged pathway from model development and internal validation through independent external validation, prospective workflow testing, assisted-use evaluation, clinical-impact assessment, and post-deployment monitoring. These stages reflect established principles of AI evaluation and provide a framework for assessing the maturity of pancreatic-surgery applications.

For predictive models, progression requires demonstration of discrimination, calibration, and clinically meaningful decision thresholds across independent populations. Prospective studies should assess data availability, reliability, latency, failure rates, and integration within clinical workflows. Assisted-use evaluations should examine usability, clinician overrides, automation bias, and unintended consequences, while impact studies should determine whether AI-informed decisions improve predefined process or patient-centred outcomes. At present, most pancreatic-resection applications remain at the development or validation stage, with relatively few undergoing prospective evaluation.

### 7.2. Prediction, Planning, and Guidance

**Figure 3:**
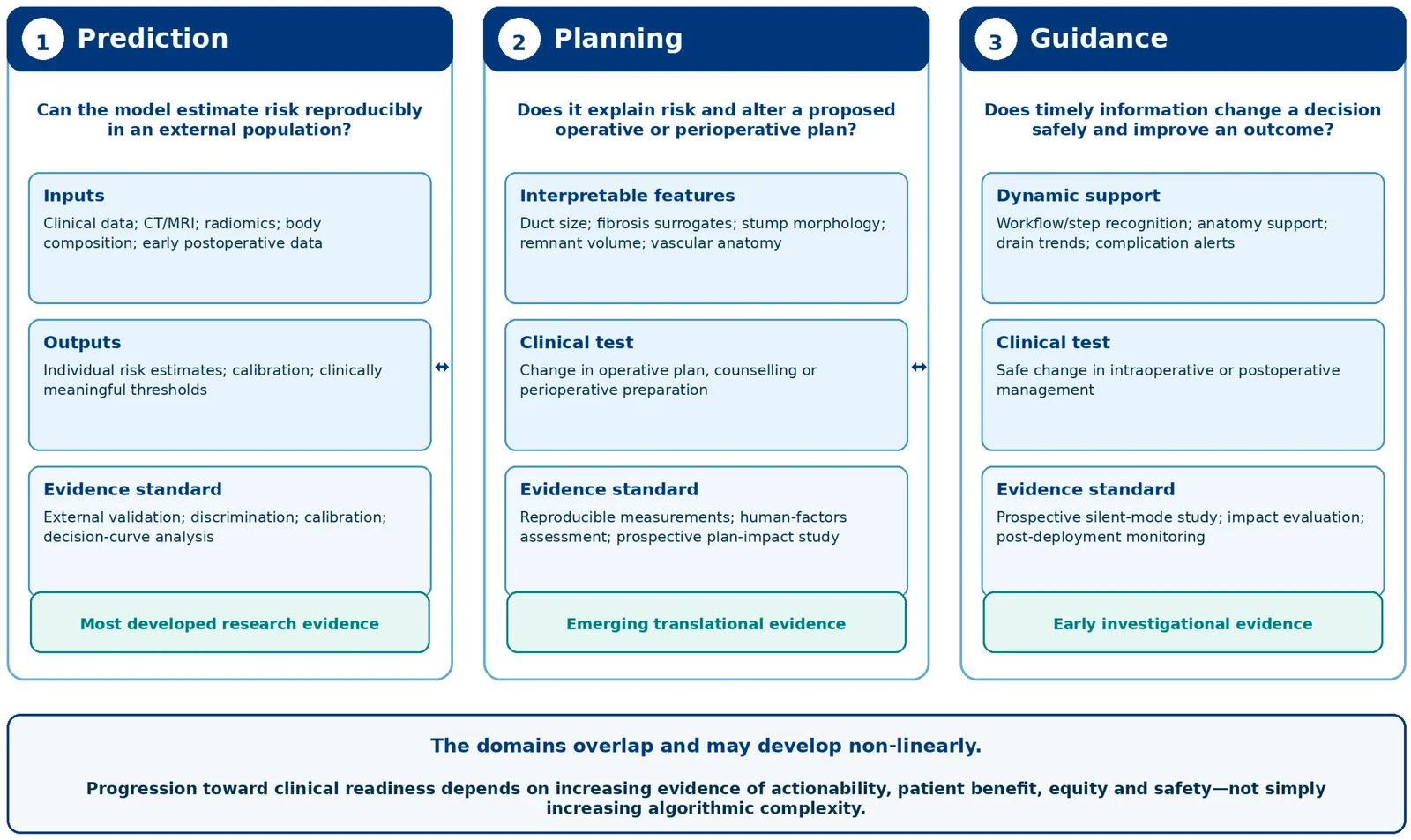
Conceptual clinical framework for artificial intelligence (AI) in pancreatic resection. Prediction, planning, and guidance represent overlapping clinical roles with different evidentiary requirements. The figure organises clinical function and does not imply readiness for routine care. Original figure.

Prediction, planning, and guidance represent distinct but overlapping clinical functions and should be considered separately from the stage of evaluation. Figure 3 is intended as a conceptual framework rather than an implementation algorithm.

Prediction: accurate, calibrated, and reproducible estimation of an outcome using variables available at the defined timepoint.

Planning: translation of preoperative anatomical or risk information into a proposed operative or perioperative strategy, with the effect on planning explicitly evaluated.

Guidance: delivery of real-time or near-real-time information that informs a clinical decision and is evaluated for its effect on decision-making, workflow, safety, or outcomes. Phase recognition or offline segmentation alone does not constitute clinical guidance.

Progress should be judged by clinical actionability, appropriate validation, and patient benefit rather than by increasing algorithmic complexity.

### 7.3. Explainability, Workflow Integration, and Human Factors

The level of explainability should be appropriate to the intended clinical decision. Interpretable outputs and measures of uncertainty may support clinician review and multidisciplinary decision-making. Future implementation studies should evaluate cognitive workload, trust, automation bias, alert fatigue, clinician overrides, and variation in performance across centres and levels of surgical experience. Intraoperative systems will also need to demonstrate reliable latency, robustness, and failure handling across representative clinical environments before routine deployment [12,64].

### 7.4. Prospective Evaluation and Clinical Impact

A key unanswered question is whether incorporating AI-generated information into clinical decision-making improves workflow, safety, or patient outcomes. Future studies should link predefined AI outputs to specific clinical actions and evaluate their impact using prospective comparative designs where appropriate. Reporting should distinguish process measures from patient-centred outcomes and include assessment of failure modes, clinician overrides, unintended consequences, equity, and resource implications.

## 8. Conclusions

Artificial intelligence (AI) applications in pancreatic resection now extend beyond clinically relevant postoperative pancreatic fistula (CR-POPF) prediction to encompass other perioperative outcomes, tissue and margin assessment, three-dimensional anatomical planning, and operative video analysis and anatomy recognition. The most mature evidence relates to CR-POPF prediction following pancreatoduodenectomy; however, external validation results remain variable, and AI models have not consistently outperformed established clinical risk scores. Evidence supporting applications in distal pancreatectomy and technical domains remains at an earlier stage of development, and no included study has yet demonstrated a clear improvement in patient-centred outcomes.

Consequently, most AI applications in pancreatic surgery remain within the phases of development, internal validation, or limited external validation. Progress towards clinical implementation will require robust independent validation, prospective workflow and human-factors evaluation, and comparative studies assessing clinical impact. AI is most likely to succeed as a decision-support tool that augments, rather than replaces, specialist surgical judgement. Its ultimate value will depend on reliable performance within real-world clinical environments and demonstrable improvements in decision-making, safety, workflow, and patient outcomes.

## Figures and Tables

**Table 1:** PubMed search concept lines.

#	Search Terms	Field
#1	“artificial intelligence” OR “machine learning” OR “deep learning” OR radiomics OR “computer vision” OR “three-dimensional reconstruction” OR “surgical video” OR “digital pathology” OR histology OR “tissue classification” OR “anatomy recognition”	Title/Abstract
#2	pancreas OR pancreatic	Title/Abstract
#3	surgery OR resection OR pancreatectomy OR pancreatoduodenectomy OR pancreaticoduodenectomy OR “distal pancreatectomy” OR “postoperative pancreatic fistula” OR POPF	Title/Abstract

The three concept lines were combined with AND. The exact final Boolean expression, including the predefined Create Date restriction, was: (“artificial intelligence”[Title/Abstract] OR “machine learning”[Title/Abstract] OR “deep learning”[Title/Abstract] OR radiomics[Title/Abstract] OR “computer vision”[Title/Abstract] OR “three dimensional reconstruction”[Title/Abstract] OR “surgical video”[Title/Abstract] OR “digital pathology”[Title/Abstract] OR histology[Title/Abstract] OR “tissue classification”[Title/Abstract] OR “anatomy recognition”[Title/Abstract]) AND (pancreas[Title/Abstract] OR pancreatic[Title/Abstract]) AND (surgery[Title/Abstract] OR resection[Title/Abstract] OR pancreatectomy[Title/Abstract] OR pancreatoduodenectomy[Title/Abstract] OR pancreaticoduodenectomy[Title/Abstract] OR “distal pancreatectomy”[Title/Abstract] OR “postoperative pancreatic fistula”[Title/Abstract] OR POPF[Title/Abstract]) AND (“1800/01/01”[CRDT]: “2026/07/24”[CRDT]).

**Table 2:** Selected representative and higher-maturity studies of pancreatic fistula prediction, with explicit information timepoint; the complete evidence inventory is provided in Supplementary Table S1.

Study	Design/Population/Operation/Outcome	Information Timepoint/Inputs/Model	Evaluation/Validation	Performance/Comparator/Calibration/Utility	Main Limitation/Maturity
Kambakamba 2020 [13]	Matched proof-of-principle; *n* = 110; PD; POPF as reported	Preoperative—non-contrast CT texture; ML	10-fold cross-validation	AUC 0.95; sensitivity 96%; specificity 98%; FRS 0.76; a-FRS 0.72; calibration/DCA NR	Selected case–control sample; optimism likely
Mu 2020 [14]	Multicentre development *n* = 513; prospective external test *n* = 70; 95/583 CR-POPF; PD; CR-POPF	Preoperative—contrast-enhanced CT; CNN score	Internal split plus external prospective test	AUC 0.85/0.81/0.89 (training/validation/test); FRS 0.78/0.76/0.73; calibration/DCA NR	Small external test; no impact study
Yoon 2022 [15]	Three-centre external cohort; *n* = 1576; PD; POPF as defined in the validation study	Mixed-timepoint validation—previously developed platform included models with preoperative and intraoperative predictors	Independent external validation	AUC 0.585–0.672; nomogram up to 0.679; calibration and threshold utility NR	External validation of mixed-timepoint platform; marked performance decline outside development centre
RAD-FRS 2023 [16]	Development in the Netherlands; external validation in Italy; *n* = 359, CR-POPF *n* = 89; PD; CR-POPF	Preoperative—CT radiomics; four ML classifiers	External-centre validation	External AUC 0.81 (95% CI 0.69–0.92); sensitivity 0.96, specificity 0.66, PPV 0.64, NPV 0.96; FRS 0.79; ua-FRS 0.79; external calibration consistent with observed risk; DCA NR	Requires CT harmonisation and further replication
Ganjouei 2023 [17]	ACS-NSQIP; *n* = 8666; CR-POPF ≈ 13%; PD; CR-POPF	Preoperative—registry variables known before surgery; LR/NN/RF/XGBoost	Internal split	Best AUC approx. 0.72; algorithms compared internally; calibration/DCA not reported in indexed record	Internal validation only; moderate discrimination; no independent external validation or impact study
Verma 2024 [18]	ACS-NSQIP *n* = 12,281; independent institutional cohort *n* = 445; PD; CR-POPF	Predominantly preoperative—clinical variables; XGBoost	Independent institutional external validation	AUC 0.72 internal and 0.79 (95% CI 0.74–0.84) external; mFRS 0.68/0.75; internal AUPRC 0.22 vs. 0.18; calibration/DCA not reported in the indexed abstract	External validation limited to one institutional cohort; no prospective impact study
Lee 2024 [19]	Single-centre temporal study; *n* = 1333; test *n* = 452; CR-POPF 6.0% in test; PD; any POPF and CR-POPF	Preoperative—clinical/body-composition ML, CT DL, and ensemble	Temporal test by surgery year	Test AUC 0.75 (95% CI 0.71–0.80) for any POPF and 0.68 (0.58–0.78) for CR-POPF; Roberts CR-POPF AUC 0.635; calibration/DCA NR	Modest CR-POPF performance
AutoFRS 2025 [20]	Development *n* = 108; external cohort *n* = 61; pancreatic-head resection; POPF as reported	Preoperative—CT plus clinical data; annotation-free multimodal DL	External cohort	Internal/external AUC 0.81/0.79; balanced accuracy 0.72/0.70; directly comparable subset: AutoFRS 0.84 vs. ua-FRS 0.85; calibration/DCA NR	Small external cohort
Liang 2025 [21]	Two institutions; DP *n* = 230; pancreatic cancer; grade B/C POPF 47.4%	Preoperative—2D/3D body composition and clinical data; DL segmentation + GBDT	External institutional test	External AUC 0.82; sensitivity 0.81; specificity 0.76; DCA used to assess net benefit; calibration NR	Cancer-specific two-centre cohort; external test *n* = 42; high event rate; tumour size and stapler use differed across cohorts
Miyamoto 2026 [22]	Single centre; DP *n* = 109; pancreatic cancer; POPF as defined by the study	Mixed-timepoint association—preoperative 3D morphometrics plus post-resection pathological fibrosis	Internal analysis	Roundness and remnant-volume ratio associated with POPF; no externally validated probability model, calibration, or decision analysis	Mixed-timepoint analysis; pathological fibrosis unavailable preoperatively; inadequate segmentations excluded; no external probability-model validation

Abbreviations: a-FRS, alternative Fistula Risk Score; AI, artificial intelligence; AUC, area under the receiver-operating-characteristic curve; AUPRC, area under the precision–recall curve; CR-POPF, clinically relevant postoperative pancreatic fistula; CT, computed tomography; DCA, decision-curve analysis; DL, deep learning; DP, distal pancreatectomy; FRS, Fistula Risk Score; GBDT, gradient-boosted decision tree; LR, logistic regression; mFRS, modified Fistula Risk Score; ML, machine learning; NN, neural network; NPV, negative predictive value; PD, pancreatoduodenectomy; PPV, positive predictive value; RF, random forest; ua-FRS, updated alternative Fistula Risk Score; XGBoost, extreme gradient boosting. NR indicates that the metric was not reported or could not be verified from the accessible indexed/full-text source used for this review.

**Table 3:** Clinical roles by task and information timepoint: demonstrated capability versus intended use.

Task/Information Timepoint	Currently Demonstrated	Potential Intended Decision	Evidence Needed Before Clinical Use	Key Implementation Barrier
Clinical prediction—preoperative	Clinical, radiomic, body-composition, and DL risk models	Counselling; baseline surveillance; trial stratification	External validation; calibration; thresholds; net benefit	Dataset shift; imaging variation; unclear action thresholds
Anatomical planning—preoperative	Pancreas/duct/vessel segmentation; remnant measurements	Approach, transection/reconstruction planning; team preparation	Prospective plan-impact study; multicentre reproducibility	Segmentation failure; manual correction; uncertain outcome effect
Tissue/margin assessment—intraoperative/post-resection	Tissue classification; margin-risk modelling; quantitative pathology	Margin assessment; biological characterisation; risk refinement	Diagnostic/workflow validation against pathology; decision-impact testing	Sampling/processing; workflow integration; generalisability
Intraoperative analysis/recognition	Phase/task recognition; vessel/duct detection; offline video analysis	Indexing/review now; future prompts or navigation	Real-time validation; latency/reliability; human factors; impact study	Annotation; deformation; false positives; centre/surgeon generalisability
Decision support—early postoperative	Dynamic models using drain amylase and clinical trajectory	Drain removal; imaging; escalation/de-escalation	Prospective protocol testing with false-negative safety endpoints	Practice variation; time-dependent data; responsibility for action
Training/technical assessment—intraoperative video	Phase segmentation and timing metrics	Feedback; learning-curve monitoring; case review	Validation against expert ratings and patient outcomes	Oversimplified quality metrics; fairness across techniques

## Data Availability

This review synthesises data from published studies cited in the manuscript. The study-selection data and evidence inventories generated for this review are provided in Supplementary Tables S1 and S2; no additional unpublished datasets were generated.

## Supplementary Materials

The following supporting information can be downloaded at: https://www.mdpi.com/article/10.3390/jcm15218737/s1, Supplementary Method S1, literature search and study selection; Supplementary Table S1, complete inventory of the 70 eligible primary reports included in the principal synthesis; Supplementary Table S2, reports excluded after detailed eligibility assessment with reasons; and Supplementary Figure S1, study-selection flow. All studies in Supplementary Table S1 are cross-referenced to the numbered bibliography; references [65,66,67,68,69,70,71,72,73,74,75,76,77,78,79,80,81,82,83] are included solely to support the supplementary evidence inventory.

## Abbreviations

The following abbreviations are used in this manuscript:

Abbreviation	Definition
3D	three-dimensional
ACS-NSQIP	American College of Surgeons National Surgical Quality Improvement Program
a-FRS	alternative Fistula Risk Score
AI	artificial intelligence
AUC	area under the receiver-operating-characteristic curve
AUPRC	area under the precision–recall curve
AUROC	area under the receiver-operating-characteristic curve
CR-POPF	clinically relevant postoperative pancreatic fistula
CT	computed tomography
DCA	decision-curve analysis
DL	deep learning
DP	distal pancreatectomy
FRS	Fistula Risk Score
GBDT	gradient-boosted decision tree
HPB	hepatopancreatobiliary
ISGPS	International Study Group of Pancreatic Surgery
LPD	laparoscopic pancreatoduodenectomy
LR	logistic regression
mFRS	modified Fistula Risk Score
ML	machine learning
MRI	magnetic resonance imaging
NN	neural network
NPV	negative predictive value
PD	pancreatoduodenectomy
POPF	postoperative pancreatic fistula
PPV	positive predictive value
RF	random forest
ua-FRS	updated alternative Fistula Risk Score
XGBoost	extreme gradient boosting
