# Supplementary material for: Artificial Intelligence in Pancreatic Resection: Current Evidence, Surgical Applications, and a Framework for Clinical Translation
Source: J Clin Med. 2026 Nov 9;15(21):8737. doi: 10.3390/jcm15218737 (PMC13764543; doi:10.3390/jcm15218737)
Supplement: Supplementary file 1 [file jcm-15-08737-s001.zip › jcm-4574477-supplementary/jcm-4574477-supplementary.pdf]

Supplementary Material

Supplementary Method S1. Literature search and study selection.

**Review design**	Focused narrative review
**Database**	PubMed/MEDLINE
**Evidence cutoff**	24 July 2026
**Evidence-cutoff restriction**	A PubMed Create Date (CRDT) restriction through 24 July 2026 was incorporated directly into the final Boolean expression to preserve the predefined evidence cutoff.
**Search-stage filters**	No language, article-type, or human-species filter was applied at search stage. English language was applied during eligibility screening.
**Eligibility**	Peer-reviewed primary human studies with pancreas-specific resection data or pancreatic-resection material/video/imaging, evaluating AI, ML, deep learning, radiomics, computer vision, AI-assisted 3D methods, or computational tissue classification with a direct perioperative or technical surgical application.
**Eligible tasks**	Resection-related complication prediction; anatomical/procedural planning; intraoperative guidance or recognition; surgical-margin assessment; early postoperative decision support.
**Principal exclusions**	Pure diagnosis/cyst classification; long-term oncological prognosis/recurrence or long-term metabolic sequelae without a direct perioperative/technical endpoint; non-pancreatic/non-separable mixed cohorts; conventional analyses without an eligible computational method; non-human studies; non-primary publications; conference abstracts/non-peer-reviewed preprints; non-English reports.
**Overlap rule**	Reports from overlapping cohorts were retained only when they addressed a distinct outcome, validation cohort, modality, or clinical application. Potential overlap is annotated in Supplementary Table S1.
**Detailed assessment**	Full text was used where available and required for interpretation; when full text was inaccessible, only explicitly indexed information was used and unreported details were not inferred.
**Exact final Boolean search**	
**Exact final Boolean search**	("artificial intelligence"[Title/Abstract] OR "machine learning"[Title/Abstract] OR "deep learning"[Title/Abstract] OR radiomics[Title/Abstract] OR "computer vision"[Title/Abstract] OR "three-dimensional reconstruction"[Title/Abstract] OR "three dimensional reconstruction"[Title/Abstract] OR "surgical video"[Title/Abstract] OR "digital pathology"[Title/Abstract] OR histology[Title/Abstract] OR "tissue classification"[Title/Abstract] OR "anatomy recognition"[Title/Abstract]) AND (pancreas[Title/Abstract] OR pancreatic[Title/Abstract]) AND (surgery[Title/Abstract] OR resection[Title/Abstract] OR pancreatectomy[Title/Abstract] OR pancreatoduodenectomy[Title/Abstract] OR pancreaticoduodenectomy[Title/Abstract] OR "distal pancreatectomy"[Title/Abstract] OR "postoperative pancreatic fistula"[Title/Abstract] OR POPF[Title/Abstract]) AND ("1800/01/01"[CRDT] : "2026/07/24"[CRDT])

Study-selection counts

Selection stage	n	Comment
PubMed records retrieved	1613	Unique PubMed records
Duplicates removed	0	PubMed PMIDs are unique
Title/abstract records screened	1613	
Excluded at title/abstract screening	1393	Outside direct pancreatic-resection AI/computational scope
Detailed eligibility assessment	220	
Excluded after detailed assessment	152	See Supplementary Table S2
Included from PubMed search	68	
Additional eligible reports retained through targeted citation-based verification	2	Bhasker 2023 and Matsui 2023
Final principal synthesis	70	See Supplementary Table S1

Supplementary Table S1. Complete inventory of eligible primary reports included in the principal synthesis.

PMID	Reference	Study	Year	Title	Primary evidence domain	Predictor / assessment timing	Computational method	Evidence maturity	Search source	Overlap / independence note	Journal	DOI	PubMed URL
37828597	[26]	Matsui	2023	A novel prediction model of pancreatic fistula after pancreaticoduodenectomy using only preoperative markers.	Clinical prediction / decision support - POPF	Preoperative	Bayesian classifier	Retrospective development / internal validation	Targeted citation-based verification	No specific overlap identified from indexed information; shared registries/institutions remain possible.	BMC Surgery	10.1186/s12893-023-02213-1	https://pubmed.ncbi.nlm.nih.gov/37828597/
37161007	[28]	Bhasker	2023	Prediction of clinically relevant postoperative pancreatic fistula using radiomic features and preoperative data.	Clinical prediction / decision support - POPF	Preoperative / Postoperative	Radiomics	Retrospective development / internal validation	Targeted citation-based verification	No specific overlap identified from indexed information; shared registries/institutions remain possible.	Scientific Reports	10.1038/s41598-023-34168-x	https://pubmed.ncbi.nlm.nih.gov/37161007/
42442221	[40]	De Robertis R	2026	CT texture analysis and delta radiomics in predicting R0 resection after preoperative chemotherapy in resectable pancreatic ductal adenocarcinoma.	Margin / tissue assessment	Preoperative / Intraoperative	Radiomics	Prospective observational / pilot	PubMed/MEDLINE search	No specific overlap identified from indexed information; shared registries/institutions remain possible.	Translational oncology	10.1016/j.tranon.2026.102900	https://pubmed.ncbi.nlm.nih.gov/42442221/
42414777	[54]	Li C	2026	Deep learning-based automatic identification model of main pancreatic duct in intraoperative ultrasonography.	Intraoperative computer vision / workflow	Intraoperative / Postoperative	Deep learning / neural network	External or multicentre evaluation	PubMed/MEDLINE search	No specific overlap identified from indexed information; shared registries/institutions remain possible.	Surgical endoscopy	10.1007/s00464-026-13056-1	https://pubmed.ncbi.nlm.nih.gov/42414777/
42335604	[42]	Fang T	2026	MICLEAR: Intelligent molecular cytology for intraoperative margin assessment of pancreatic ductal adenocarcinoma.	Margin / tissue assessment	Intraoperative	Computational model	Retrospective development / internal validation	PubMed/MEDLINE search	No specific overlap identified from indexed information; shared registries/institutions remain possible.	Medical image analysis	10.1016/j.media.2026.104181	https://pubmed.ncbi.nlm.nih.gov/42335604/
42115020	[50]	Takayama M	2026	Artificial intelligence-assisted estimation of superior mesenteric artery dissection extent during robotic pancreatoduodenectomy.	Intraoperative computer vision / workflow	Intraoperative / Postoperative	Artificial intelligence (unspecified)	Technical feasibility / model validation	PubMed/MEDLINE search	No specific overlap identified from indexed information; shared registries/institutions remain possible.	HPB : the official journal of the International Hepato Pancreato Biliary Association	10.1016/j.hpb.2026.04.455	https://pubmed.ncbi.nlm.nih.gov/42115020/
42070929	[39]	Ricker AB	2026	Deep learning via pathology-image integration for margin prediction after pancreatoduodenectomy.	Margin / tissue assessment	Preoperative / Intraoperative	Deep learning / neural network	Prospective observational / pilot	PubMed/MEDLINE search	No specific overlap identified from indexed information; shared registries/institutions remain possible.	HPB : the official journal of the International Hepato Pancreato Biliary Association	10.1016/j.hpb.2026.04.445	https://pubmed.ncbi.nlm.nih.gov/42070929/
41973289	[51]	Wakabayashi T	2026	Robotic Pancreaticoduodenectomy with Right Lateral Approach to the Superior Mesenteric Artery: Surgical Artificial Intelligence-Based Visualization for Standardized Dissection.	Intraoperative computer vision / workflow	Intraoperative	Computer vision	Case report / proof of concept	PubMed/MEDLINE search	No specific overlap identified from indexed information; shared registries/institutions remain possible.	Annals of surgical oncology	10.1245/s10434-026-19617-x	https://pubmed.ncbi.nlm.nih.gov/41973289/
41895782	[65]	Matsuda K	2026	Usefulness of Artificial Intelligence for Surgical Support in Robot-assisted Distal Pancreatectomy: A Preliminary Case Report.	Intraoperative computer vision / workflow	Intraoperative / Postoperative	Computer vision	Case report / proof of concept	PubMed/MEDLINE search	No specific overlap identified from indexed information; shared registries/institutions remain possible.	Anticancer research	10.21873/anticanres.18117	https://pubmed.ncbi.nlm.nih.gov/41895782/
41723320	[22]	Miyamoto R	2026	Prediction of Postoperative Pancreatic Fistula Occurrence by the Pancreatic Stump Shape and Remnant Pancreatic Volume Measured Using AI-Guided Three-Dimensional Simulation.	Clinical prediction / decision support - POPF	Preoperative / Postoperative	3D modelling/segmentation	Retrospective development / internal validation	PubMed/MEDLINE search	Miyamoto/SYNAPSE VINCENT programme; distinct stump-shape/remnant-volume outcome study.	Annals of surgical oncology	10.1245/s10434-026-19267-z	https://pubmed.ncbi.nlm.nih.gov/41723320/
41704439	[66]	Zhou H	2026	Construction and validation of a high-precision annotated dataset for developing intelligent critical vein recognition models in laparoscopic pancreatic surgery.	Intraoperative computer vision / workflow	Intraoperative	Computer vision	Technical feasibility / model validation	PubMed/MEDLINE search	PUMCH video dataset/benchmark; possible overlap with Shi 2025 institutional video source; retained as distinct dataset-validation contribution.	Frontiers in surgery	10.3389/fsurg.2026.1711392	https://pubmed.ncbi.nlm.nih.gov/41704439/
41673079	[67]	Garnier J	2026	Preoperative lymphocyte signature predicts pancreatic fistula after pancreatoduodenectomy.	Clinical prediction / decision support - POPF	Preoperative / Postoperative	Computational model	Prospective observational / pilot	PubMed/MEDLINE search	No specific overlap identified from indexed information; shared registries/institutions remain possible.	Communications medicine	10.1038/s43856-026-01422-y	https://pubmed.ncbi.nlm.nih.gov/41673079/
41307673	[48]	Bohm D	2026	MKNet-family architectures for auto-segmentation of the residual pancreas after pancreatic resection: a deep learning comparative study.	Anatomical segmentation / 3D planning	Preoperative / Postoperative	Deep learning / neural network	Technical feasibility / model validation	PubMed/MEDLINE search	No specific overlap identified from indexed information; shared registries/institutions remain possible.	Abdominal radiology (New York)	10.1007/s00261-025-05211-4	https://pubmed.ncbi.nlm.nih.gov/41307673/
41251913	[45]	Mellado S	2026	Deep-Learning Virtual Superior Mesenteric Artery Modeling for Risk Stratification in Pancreas Surgery.	Anatomical segmentation / 3D planning	Preoperative / Intraoperative / Postoperative	Deep learning / neural network; 3D modelling/segmentation	Technical feasibility / model validation	PubMed/MEDLINE search	No specific overlap identified from indexed information; shared registries/institutions remain possible.	Annals of surgical oncology	10.1245/s10434-025-18543-8	https://pubmed.ncbi.nlm.nih.gov/41251913/
41186562	[63]	Winter A	2026	Overcoming the data barrier: transfer learning for 90-day mortality prediction in general surgery - a retrospective multicenter development and comparison study.	Clinical prediction / guidance - other perioperative outcomes	Preoperative	Deep learning / neural network; Machine learning	External or multicentre evaluation	PubMed/MEDLINE search	Likely partial institutional overlap with Riepe 2025; retained because this is an expanded three-centre transfer-learning evaluation with distinct methodology.	International journal of surgery (London, England)	10.1097/JS9.0000000000003595	https://pubmed.ncbi.nlm.nih.gov/41186562/
39176476	[68]	Wang J	2026	A Computer Vision Algorithm to Predict Superior Mesenteric Artery Margin Status for Patients With Pancreatic Ductal Adenocarcinoma.	Margin / tissue assessment	Preoperative / Intraoperative	Computer vision	Technical feasibility / model validation	PubMed/MEDLINE search	No specific overlap identified from indexed information; shared registries/institutions remain possible.	Annals of surgery	10.1097/SLA.0000000000006506	https://pubmed.ncbi.nlm.nih.gov/39176476/
41514543	[46]	Hu T	2025	Development and Validation of a CT Radiomics-Deep Learning Model for Predicting Surgical Difficulty in Pancreatic and Periampullary Tumors.	Clinical prediction / guidance - other perioperative outcomes	Preoperative / Intraoperative	Deep learning / neural network; Machine learning; Radiomics	Retrospective development / internal validation	PubMed/MEDLINE search	No specific overlap identified from indexed information; shared registries/institutions remain possible.	Cancers	10.3390/cancers18010029	https://pubmed.ncbi.nlm.nih.gov/41514543/
41340556	[21]	Liang K	2025	Integrative deep learning analysis of 2D and 3D body composition features for predicting postoperative pancreatic fistula after distal pancreatectomy.	Clinical prediction / decision support - POPF	Preoperative / Postoperative	Deep learning / neural network; 3D modelling/segmentation	External or multicentre evaluation	PubMed/MEDLINE search	No specific overlap identified from indexed information; shared registries/institutions remain possible.	Annals of medicine	10.1080/07853890.2025.2597067	https://pubmed.ncbi.nlm.nih.gov/41340556/

PMID	Reference	Study	Year	Title	Primary evidence domain	Predictor / assessment timing	Computational method	Evidence maturity	Search source	Overlap / independence note	Journal	DOI	PubMed URL
41310921	[32]	Choubey AP	2025	Postoperative Pancreatic Fistula After Pancreatoduodenectomy: Can Radiomics Improve Clinical Risk Scores?	Clinical prediction / decision support - POPF	Preoperative / Intraoperative / Postoperative	Machine learning; Radiomics	Prospective observational / pilot	PubMed/MEDLINE search	No specific overlap identified from indexed information; shared registries/institutions remain possible.	Annals of surgery	10.1097/SLA.0000000000006991	https://pubmed.ncbi.nlm.nih.gov/41310921/
41300859	[69]	Kim YJ	2025	Deep Learning Model for Volume Measurement of the Remnant Pancreas After Pancreaticoduodenectomy and Distal Pancreatectomy.	Anatomical segmentation / 3D planning	Postoperative	Deep learning / neural network; 3D modelling/segmentation	Technical feasibility / model validation	PubMed/MEDLINE search	No specific overlap identified from indexed information; shared registries/institutions remain possible.	Diagnostics (Basel, Switzerland)	10.3390/diagnostics15222834	https://pubmed.ncbi.nlm.nih.gov/41300859/
41087962	[33]	Yan P	2025	Machine learning model based on preoperative MRI and clinical data for predicting pancreatic fistula after pancreaticoduodenectomy.	Clinical prediction / decision support - POPF	Preoperative / Postoperative	Machine learning; Radiomics	Retrospective development / internal validation	PubMed/MEDLINE search	No specific overlap identified from indexed information; shared registries/institutions remain possible.	BMC medical imaging	10.1186/s12880-025-01951-3	https://pubmed.ncbi.nlm.nih.gov/41087962/
40669905	[60]	Zhang Z	2025	Machine learning model for postpancreaticoduodenectomy haemorrhage prediction: an international multicentre cohort study.	Clinical prediction / guidance - other perioperative outcomes	Mixed/unclear	Machine learning	External or multicentre evaluation	PubMed/MEDLINE search	NSQIP training plus independent Chinese external validation for PPH; distinct outcome.	BMJ open	10.1136/bmjopen-2024-096147	https://pubmed.ncbi.nlm.nih.gov/40669905/
40585479	[70]	Zhang K	2025	Risk prediction and clinical utility analysis of postoperative pancreatic fistula: a comparative study of multivariable logistic regression and random forest models.	Clinical prediction / decision support - POPF	Postoperative	Machine learning	Retrospective development / internal validation	PubMed/MEDLINE search	No specific overlap identified from indexed information; shared registries/institutions remain possible.	Frontiers in surgery	10.3389/fsurg.2025.1596224	https://pubmed.ncbi.nlm.nih.gov/40585479/
40576127	[44]	Rhu J	2025	3D auto-segmentation of pancreas cancer and surrounding anatomical structures for surgical planning.	Anatomical segmentation / 3D planning	Preoperative	Deep learning / neural network; Computer vision; 3D modelling/segmentation	External or multicentre evaluation	PubMed/MEDLINE search	No specific overlap identified from indexed information; shared registries/institutions remain possible.	International journal of surgery (London, England)	10.1097/JS9.0000000000002835	https://pubmed.ncbi.nlm.nih.gov/40576127/
40507327	[56]	Cammarata R	2025	Machine Learning for Predicting the Low Risk of Postoperative Pancreatic Fistula After Pancreaticoduodenectomy: Toward a Dynamic and Personalized Postoperative Management Strategy.	Clinical prediction / decision support - POPF	Postoperative	Machine learning	External or multicentre evaluation	PubMed/MEDLINE search	No specific overlap identified from indexed information; shared registries/institutions remain possible.	Cancers	10.3390/cancers17111846	https://pubmed.ncbi.nlm.nih.gov/40507327/
40312536	[53]	Shi J	2025	Deep learning HRNet FCN for blood vessel identification in laparoscopic pancreatic surgery.	Intraoperative computer vision / workflow	Intraoperative	Deep learning / neural network; Computer vision	Technical feasibility / model validation	PubMed/MEDLINE search	PUMCH laparoscopic pancreatic-surgery video programme; possible overlap with Zhou 2026 dataset work; retained for distinct model evaluation.	NPJ digital medicine	10.1038/s41746-025-01663-6	https://pubmed.ncbi.nlm.nih.gov/40312536/
40307833	[31]	Wang Q	2025	Machine learning-based prediction of postoperative pancreatic fistula after laparoscopic pancreaticoduodenectomy.	Clinical prediction / decision support - POPF	Preoperative / Postoperative	Machine learning	Retrospective development / internal validation	PubMed/MEDLINE search	No specific overlap identified from indexed information; shared registries/institutions remain possible.	BMC surgery	10.1186/s12893-025-02935-4	https://pubmed.ncbi.nlm.nih.gov/40307833/
40256777	[30]	Li Y	2025	Enhanced preoperative prediction of pancreatic fistula using radiomics and clinical features with SHAP visualization.	Clinical prediction / decision support - POPF	Preoperative / Postoperative	Machine learning; Radiomics	Technical feasibility / model validation	PubMed/MEDLINE search	No specific overlap identified from indexed information; shared registries/institutions remain possible.	Frontiers in bioengineering and biotechnology	10.3389/fbioe.2025.1510642	https://pubmed.ncbi.nlm.nih.gov/40256777/
40235910	[71]	Potievskiy MB	2025	Machine learning for modeling and identifying risk factors of pancreatic fistula.	Clinical prediction / decision support - POPF	Intraoperative / Postoperative	Machine learning	Retrospective development / internal validation	PubMed/MEDLINE search	No specific overlap identified from indexed information; shared registries/institutions remain possible.	World journal of gastrointestinal oncology	10.4251/wjgo.v17.i4.100089	https://pubmed.ncbi.nlm.nih.gov/40235910/
40171562	[62]	Riepe C	2025	90-day mortality prediction in elective visceral surgery using machine learning: a retrospective multicenter development, validation, and comparison study.	Clinical prediction / guidance - other perioperative outcomes	Preoperative	Machine learning	External or multicentre evaluation	PubMed/MEDLINE search	Likely partial institutional overlap with Winter 2026; retained as the earlier multicentre organ-specific vs aggregated-model comparison.	International journal of surgery (London, England)	10.1097/JS9.0000000000002372	https://pubmed.ncbi.nlm.nih.gov/40171562/
40146236	[20]	Kolbinger FR	2025	AutoFRS: an externally validated, annotation-free approach to computational preoperative complication risk stratification in pancreatic surgery - an experimental study.	Clinical prediction / decision support - POPF	Preoperative	Deep learning / neural network; multimodal CT + clinical data	External or multicentre evaluation	PubMed/MEDLINE search	Annotation-free multimodal model; related research network to prior radiomics work; retained as distinct method/external evaluation.	International journal of surgery (London, England)	10.1097/JS9.0000000000002327	https://pubmed.ncbi.nlm.nih.gov/40146236/
40062328	[61]	Ma JM	2025	Machine learning model-based prediction of postpancreatectomy acute pancreatitis following pancreaticoduodenectomy: A retrospective cohort study.	Clinical prediction / guidance - other perioperative outcomes	Postoperative	Machine learning	Retrospective development / internal validation	PubMed/MEDLINE search	No specific overlap identified from indexed information; shared registries/institutions remain possible.	World journal of gastroenterology	10.3748/wjg.v31.i8.102071	https://pubmed.ncbi.nlm.nih.gov/40062328/
39979688	[59]	Ahmed KS	2025	Machine Learning-Driven Modeling to Predict Postdischarge Venous Thromboembolism After Pancreatectomy for Pancreas Cancer.	Clinical prediction / guidance - other perioperative outcomes	Intraoperative / Postoperative	Machine learning	Retrospective development / internal validation	PubMed/MEDLINE search	NSQIP pancreatectomy cohort for post-discharge VTE; distinct outcome.	Annals of surgical oncology	10.1245/s10434-025-17032-2	https://pubmed.ncbi.nlm.nih.gov/39979688/
39818518	[35]	Malyi A	2025	Postoperative pancreatic fistula risk assessment using digital pathology based analyses at the parenchymal resection margin of the pancreas - Results from the randomized multicenter RECOPANC trial.	Clinical prediction / decision support - POPF	Postoperative	Machine learning; Digital/computational pathology	External or multicentre evaluation	PubMed/MEDLINE search	No specific overlap identified from indexed information; shared registries/institutions remain possible.	HPB : the official journal of the International Hepato Pancreato Biliary Association	10.1016/j.hpb.2024.12.012	https://pubmed.ncbi.nlm.nih.gov/39818518/
36803027	[72]	Zheng J	2025	Development of a Pancreatic Fistula Prediction Model After Pancreaticoduodenectomy Based on a Decision Tree and Random Forest Algorithm.	Clinical prediction / decision support - POPF	Postoperative	Machine learning; Decision tree / recursive partitioning	Retrospective development / internal validation	PubMed/MEDLINE search	No specific overlap identified from indexed information; shared registries/institutions remain possible.	The American surgeon	10.1177/00031348231158692	https://pubmed.ncbi.nlm.nih.gov/36803027/
39774499	[73]	Bonde M	2024	Assessing the value of deep neural networks for postoperative complication prediction in pancreaticoduodenectomy patients.	Clinical prediction / guidance - other perioperative outcomes	Postoperative	Deep learning / neural network; Machine learning	Retrospective development / internal validation	PubMed/MEDLINE search	NSQIP-based PD complication modelling; registry overlap possible with other NSQIP reports, but transfer-learning question differs.	PloS one	10.1371/journal.pone.0316402	https://pubmed.ncbi.nlm.nih.gov/39774499/

PMID	Reference	Study	Year	Title	Primary evidence domain	Predictor / assessment timing	Computational method	Evidence maturity	Search source	Overlap / independence note	Journal	DOI	PubMed URL
39421053	[29]	Wu H	2024	Computed tomography-based radiomics and body composition analysis for predicting clinically relevant postoperative pancreatic fistula after pancreaticoduodenectomy.	Clinical prediction / decision support - POPF	Preoperative / Postoperative	Radiomics	Retrospective development / internal validation	PubMed/MEDLINE search	No specific overlap identified from indexed information; shared registries/institutions remain possible.	Gland surgery	10.21037/gs-24-167	https://pubmed.ncbi.nlm.nih.gov/39421053/
38958719	[52]	You J	2024	Artificial intelligence automated surgical phases recognition in intraoperative videos of laparoscopic pancreatoduodenectomy.	Intraoperative computer vision / workflow	Intraoperative	Deep learning / neural network	Technical feasibility / model validation	PubMed/MEDLINE search	No specific overlap identified from indexed information; shared registries/institutions remain possible.	Surgical endoscopy	10.1007/s00464-024-10916-6	https://pubmed.ncbi.nlm.nih.gov/38958719/
38873261	[74]	Wang H	2024	Guiding post-pancreaticoduodenectomy interventions for pancreatic cancer patients utilizing decision tree models.	Clinical prediction / guidance - other perioperative outcomes	Postoperative	Decision tree / recursive partitioning	Retrospective development / internal validation	PubMed/MEDLINE search	No specific overlap identified from indexed information; shared registries/institutions remain possible.	Frontiers in oncology	10.3389/fonc.2024.1399297	https://pubmed.ncbi.nlm.nih.gov/38873261/
38815516	[58]	Galouzis N	2024	Pilot Study to Optimize Goal-directed Hemodynamic Management During Pancreatectomy.	Clinical prediction / guidance - other perioperative outcomes	Intraoperative	Machine learning	Retrospective development / internal validation	PubMed/MEDLINE search	No specific overlap identified from indexed information; shared registries/institutions remain possible.	The Journal of surgical research	10.1016/j.jss.2024.04.035	https://pubmed.ncbi.nlm.nih.gov/38815516/
38601417	[47]	Miyamoto R	2024	Laparoscopic Distal Pancreatectomy Using Three-Dimensional Computer Graphics for Surgical Navigation With a Deep Learning Algorithm: A Case Report.	Anatomical segmentation / 3D planning	Preoperative / Intraoperative	Deep learning / neural network; 3D modelling/segmentation	Case report / proof of concept	PubMed/MEDLINE search	Miyamoto/SYNAPSE VINCENT programme; single-case surgical-navigation application, not efficacy evidence.	Cureus	10.7759/cureus.55907	https://pubmed.ncbi.nlm.nih.gov/38601417/
38488870	[49]	Al Abbas AI	2024	The development of a deep learning model for automated segmentation of the robotic pancreaticojejunostomy.	Intraoperative computer vision / workflow	Intraoperative / Postoperative	Computer vision; deep learning / neural network	Technical feasibility / model validation	PubMed/MEDLINE search	No specific overlap identified from indexed information; shared registries/institutions remain possible.	Surgical endoscopy	10.1007/s00464-024-10725-x	https://pubmed.ncbi.nlm.nih.gov/38488870/
38455348	[75]	Dai Y	2024	Machine learning models and nomogram based on clinical, laboratory profiles and skeletal muscle index to predict pancreatic fistula after pancreatoduodenectomy.	Clinical prediction / decision support - POPF	Postoperative	Machine learning	Retrospective development / internal validation	PubMed/MEDLINE search	No specific overlap identified from indexed information; shared registries/institutions remain possible.	Gland surgery	10.21037/gs-23-410	https://pubmed.ncbi.nlm.nih.gov/38455348/
38429308	[19]	Lee W	2024	Deep learning-based prediction of post-pancreaticoduodenectomy pancreatic fistula.	Clinical prediction / decision support - POPF	Preoperative / Postoperative	Deep learning / neural network; Machine learning	Technical feasibility / model validation	PubMed/MEDLINE search	No specific overlap identified from indexed information; shared registries/institutions remain possible.	Scientific reports	10.1038/s41598-024-51777-2	https://pubmed.ncbi.nlm.nih.gov/38429308/
38151623	[38]	Wang K	2024	Delta Radiomic Features Predict Resection Margin Status and Overall Survival in Neoadjuvant-Treated Pancreatic Cancer Patients.	Margin / tissue assessment	Preoperative / Intraoperative	Radiomics	External or multicentre evaluation	PubMed/MEDLINE search	No specific overlap identified from indexed information; shared registries/institutions remain possible.	Annals of surgical oncology	10.1245/s10434-023-14805-5	https://pubmed.ncbi.nlm.nih.gov/38151623/
37947154	[18]	Verma A	2024	Machine Learning-based Prediction of Postoperative Pancreatic Fistula Following Pancreaticoduodenectomy.	Clinical prediction / decision support - POPF	Postoperative	Machine learning	External or multicentre evaluation	PubMed/MEDLINE search	NSQIP development plus independent institutional external validation; registry overlap possible with other NSQIP development reports.	Annals of surgery	10.1097/SLA.0000000000006123	https://pubmed.ncbi.nlm.nih.gov/37947154/
37927251	[76]	Huang YL	2024	Preoperative ultrasound radiomics for predicting clinically relevant postoperative pancreatic fistula after pancreatectomy.	Clinical prediction / decision support - POPF	Preoperative / Postoperative	Radiomics	Prospective observational / pilot	PubMed/MEDLINE search	No specific overlap identified from indexed information; shared registries/institutions remain possible.	Clinical hemorheology and microcirculation	10.3233/CH-231955	https://pubmed.ncbi.nlm.nih.gov/37927251/
37811791	[16]	Ingwersen EW	2023	Radiomics preoperative-Fistula Risk Score (RAD-FRS) for pancreatoduodenectomy: development and external validation.	Clinical prediction / decision support - POPF	Preoperative / Postoperative	Machine learning; Radiomics	External or multicentre evaluation	PubMed/MEDLINE search	RAD-FRS development/external-validation programme; related centres/research network to later annotation-free models may overlap.	BJS open	10.1093/bjsopen/zrad100	https://pubmed.ncbi.nlm.nih.gov/37811791/
37550449	[17]	Ashraf Ganjouei A	2023	A Machine Learning Approach to Predict Postoperative Pancreatic Fistula After Pancreaticoduodenectomy Using Only Preoperatively Known Data.	Clinical prediction / decision support - POPF	Preoperative / Intraoperative / Postoperative	Deep learning / neural network; Machine learning	Technical feasibility / model validation	PubMed/MEDLINE search	ACS-NSQIP POPF model; registry overlap possible with other NSQIP-based reports.	Annals of surgical oncology	10.1245/s10434-023-14041-x	https://pubmed.ncbi.nlm.nih.gov/37550449/
37150712	[34]	Ingwersen EW	2023	Machine learning versus logistic regression for the prediction of complications after pancreatoduodenectomy.	Clinical prediction / guidance - other perioperative outcomes	Postoperative	Machine learning	Retrospective development / internal validation	PubMed/MEDLINE search	No specific overlap identified from indexed information; shared registries/institutions remain possible.	Surgery	10.1016/j.surg.2023.03.012	https://pubmed.ncbi.nlm.nih.gov/37150712/
36306322	[43]	Miyamoto R	2022	Three-dimensional simulation of the pancreatic parenchyma, pancreatic duct and vascular arrangement in pancreatic surgery using a deep learning algorithm.	Anatomical segmentation / 3D planning	Preoperative	Deep learning / neural network; 3D modelling/segmentation	Retrospective development / internal validation	PubMed/MEDLINE search	Miyamoto/SYNAPSE VINCENT programme; distinct technical segmentation study.	PloS one	10.1371/journal.pone.0276600	https://pubmed.ncbi.nlm.nih.gov/36306322/
36185559	[77]	Long ZD	2022	Personal predictive model based on systemic inflammation markers for estimation of postoperative pancreatic fistula following pancreaticoduodenectomy.	Clinical prediction / decision support - POPF	Postoperative	Deep learning / neural network; Machine learning	Retrospective development / internal validation	PubMed/MEDLINE search	No specific overlap identified from indexed information; shared registries/institutions remain possible.	World journal of gastrointestinal surgery	10.4240/wjgs.v14.i9.963	https://pubmed.ncbi.nlm.nih.gov/36185559/
35508684	[78]	Chen KA	2022	Differential Performance of Machine Learning Models in Prediction of Procedure-Specific Outcomes.	Clinical prediction / guidance - other perioperative outcomes	Intraoperative	Deep learning / neural network; Machine learning	Technical feasibility / model validation	PubMed/MEDLINE search	NSQIP-based procedure-specific modelling; registry overlap possible with other NSQIP reports, but outcome/model question differs.	Journal of gastrointestinal surgery : official journal of the Society for Surgery of the Alimentary Tract	10.1007/s11605-022-05332-x	https://pubmed.ncbi.nlm.nih.gov/35508684/
35500881	[55]	Shen Z	2022	Machine learning algorithms as early diagnostic tools for pancreatic fistula following pancreaticoduodenectomy and guide drain removal: A retrospective cohort study.	Clinical prediction / decision support - POPF	Preoperative / Intraoperative / Postoperative	Machine learning	Technical feasibility / model validation	PubMed/MEDLINE search	No specific overlap identified from indexed information; shared registries/institutions remain possible.	International journal of surgery (London, England)	10.1016/j.ijsu.2022.106638	https://pubmed.ncbi.nlm.nih.gov/35500881/
35317357	[15]	Yoon SJ	2022	External validation of risk prediction platforms for pancreatic fistula after pancreatoduodenectomy using nomograms and artificial intelligence.	Clinical prediction / decision support - POPF	Postoperative	Artificial intelligence (unspecified)	External or multicentre evaluation	PubMed/MEDLINE search	External validation of Han 2020 platform; retained as a distinct external-validation report.	Annals of surgical treatment and research	10.4174/astr.2022.102.3.147	https://pubmed.ncbi.nlm.nih.gov/35317357/

PMID	Reference	Study	Year	Title	Primary evidence domain	Predictor / assessment timing	Computational method	Evidence maturity	Search source	Overlap / independence note	Journal	DOI	PubMed URL
34596836	[27]	Capretti G	2022	A machine learning risk model based on preoperative computed tomography scan to predict postoperative outcomes after pancreatoduodenectomy.	Clinical prediction / decision support - POPF	Preoperative	Machine learning; Radiomics	Retrospective development / internal validation	PubMed/MEDLINE search	No specific overlap identified from indexed information; shared registries/institutions remain possible.	Updates in surgery	10.1007/s13304-021-01174-5	https://pubmed.ncbi.nlm.nih.gov/34596836/
34891726	[79]	Pfitzner B	2021	Perioperative Risk Assessment in Pancreatic Surgery Using Machine Learning.	Clinical prediction / guidance - other perioperative outcomes	Postoperative	Machine learning	Retrospective development / internal validation	PubMed/MEDLINE search	Charite pancreatic-resection cohort; related research programme to later Riepe/Winter work but distinct perioperative dynamic-risk application.	Annual International Conference of the IEEE Engineering in Medicine and Biology Society. IEEE Engineering in Medicine and Biology Society. Annual International Conference	10.1109/EMBC46164.2021.9630897	https://pubmed.ncbi.nlm.nih.gov/34891726/
34752505	[80]	Wakiya T	2021	Prediction of massive bleeding in pancreatic surgery based on preoperative patient characteristics using a decision tree.	Clinical prediction / guidance - other perioperative outcomes	Preoperative / Intraoperative	Machine learning; Decision tree / recursive partitioning	Retrospective development / internal validation	PubMed/MEDLINE search	No specific overlap identified from indexed information; shared registries/institutions remain possible.	PloS one	10.1371/journal.pone.0259682	https://pubmed.ncbi.nlm.nih.gov/34752505/
33945924	[24]	Skawran SM	2021	Can magnetic resonance imaging radiomics of the pancreas predict postoperative pancreatic fistula?	Clinical prediction / decision support - POPF	Postoperative	Machine learning; Radiomics	Technical feasibility / model validation	PubMed/MEDLINE search	No specific overlap identified from indexed information; shared registries/institutions remain possible.	European journal of radiology	10.1016/j.ejrad.2021.109733	https://pubmed.ncbi.nlm.nih.gov/33945924/
33857829	[81]	Lin Z	2021	Preoperative prediction of clinically relevant postoperative pancreatic fistula after pancreaticoduodenectomy.	Clinical prediction / decision support - POPF	Preoperative / Postoperative	Radiomics; 3D modelling/segmentation	Technical feasibility / model validation	PubMed/MEDLINE search	No specific overlap identified from indexed information; shared registries/institutions remain possible.	European journal of radiology	10.1016/j.ejrad.2021.109693	https://pubmed.ncbi.nlm.nih.gov/33857829/
33770412	[82]	Giovinazzo F	2021	Clinical relevant pancreatic fistula after pancreatoduodenectomy: when negative amylase levels tell the truth.	Clinical prediction / decision support - POPF	Postoperative	Machine learning	External or multicentre evaluation	PubMed/MEDLINE search	No specific overlap identified from indexed information; shared registries/institutions remain possible.	Updates in surgery	10.1007/s13304-021-01020-8	https://pubmed.ncbi.nlm.nih.gov/33770412/
33734096	[57]	Cos H	2021	Predicting Outcomes in Patients Undergoing Pancreatectomy Using Wearable Technology and Machine Learning: Prospective Cohort Study.	Clinical prediction / guidance - other perioperative outcomes	Mixed/unclear	Machine learning	Prospective observational / pilot	PubMed/MEDLINE search	No specific overlap identified from indexed information; shared registries/institutions remain possible.	Journal of medical Internet research	10.2196/23595	https://pubmed.ncbi.nlm.nih.gov/33734096/
32874057	[25]	Han IW	2020	Risk prediction platform for pancreatic fistula after pancreatoduodenectomy using artificial intelligence.	Clinical prediction / decision support - POPF	Preoperative / Intraoperative / Postoperative	Deep learning / neural network; Machine learning	Retrospective development / internal validation	PubMed/MEDLINE search	Development report for AI POPF platform subsequently externally validated by Yoon 2022.	World journal of gastroenterology	10.3748/wjg.v26.i30.4453	https://pubmed.ncbi.nlm.nih.gov/32874057/
32863959	[14]	Mu W	2020	Prediction of clinically relevant Pancreatico-enteric Anastomotic Fistulas after Pancreatoduodenectomy using deep learning of Preoperative Computed Tomography.	Clinical prediction / decision support - POPF	Preoperative / Intraoperative / Postoperative	Deep learning / neural network	Prospective observational / pilot	PubMed/MEDLINE search	No specific overlap identified from indexed information; shared registries/institutions remain possible.	Theranostics	10.7150/thno.49671	https://pubmed.ncbi.nlm.nih.gov/32863959/
32377222	[36]	Hui B	2020	Identification of Pancreaticoduodenectomy Resection for Pancreatic Head Adenocarcinoma: A Preliminary Study of Radiomics.	Margin / tissue assessment	Preoperative / Intraoperative	Machine learning; Radiomics; Digital/computational pathology	Retrospective development / internal validation	PubMed/MEDLINE search	No specific overlap identified from indexed information; shared registries/institutions remain possible.	Computational and mathematical methods in medicine	10.1155/2020/2761627	https://pubmed.ncbi.nlm.nih.gov/32377222/
31932878	[37]	Bian Y	2020	Performance of CT-based radiomics in diagnosis of superior mesenteric vein resection margin in patients with pancreatic head cancer.	Margin / tissue assessment	Intraoperative / Postoperative	Radiomics	Retrospective development / internal validation	PubMed/MEDLINE search	No specific overlap identified from indexed information; shared registries/institutions remain possible.	Abdominal radiology (New York)	10.1007/s00261-019-02401-9	https://pubmed.ncbi.nlm.nih.gov/31932878/
31727325	[13]	Kambakamba P	2020	The potential of machine learning to predict postoperative pancreatic fistula based on preoperative, non-contrast-enhanced CT: A proof-of-principle study.	Clinical prediction / decision support - POPF	Preoperative / Intraoperative / Postoperative	Machine learning	Prospective observational / pilot	PubMed/MEDLINE search	No specific overlap identified from indexed information; shared registries/institutions remain possible.	Surgery	10.1016/j.surg.2019.09.019	https://pubmed.ncbi.nlm.nih.gov/31727325/
30568506	[23]	Zhang W	2018	A radiomics-based formula for the preoperative prediction of postoperative pancreatic fistula in patients with pancreaticoduodenectomy.	Clinical prediction / decision support - POPF	Preoperative / Postoperative	Radiomics	Retrospective development / internal validation	PubMed/MEDLINE search	No specific overlap identified from indexed information; shared registries/institutions remain possible.	Cancer management and research	10.2147/CMAR.S185865	https://pubmed.ncbi.nlm.nih.gov/30568506/
27575375	[41]	Eberlin LS	2016	Pancreatic Cancer Surgical Resection Margins: Molecular Assessment by Mass Spectrometry Imaging.	Margin / tissue assessment	Intraoperative	Digital/computational pathology; Mass-spectrometry imaging + LASSO classifier	Prospective observational / pilot	PubMed/MEDLINE search	No specific overlap identified from indexed information; shared registries/institutions remain possible.	PLoS medicine	10.1371/journal.pmed.1002108	https://pubmed.ncbi.nlm.nih.gov/27575375/
27046794	[83]	Jutric Z	2016	Preoperative computed tomography scan to predict pancreatic fistula after distal pancreatectomy using gland and tumor characteristics.	Clinical prediction / decision support - POPF	Preoperative / Postoperative	Decision tree / recursive partitioning	External or multicentre evaluation	PubMed/MEDLINE search	No specific overlap identified from indexed information; shared registries/institutions remain possible.	American journal of surgery	10.1016/j.amjsurg.2016.02.001	https://pubmed.ncbi.nlm.nih.gov/27046794/

Supplementary Table S2. Reports excluded after detailed eligibility assessment, with the specific reason for exclusion.

PMID	Study	Year	Title	Detailed-assessment exclusion category	Specific exclusion reason	Publication type	Language	Journal	DOI	PubMed URL
41801453	Palecek V	2026	Correlation between inflammation, vascularization, adipocyte percentage and exocrine cell percentage at the resection margin and the postoperative pancreatic fistula rate after distal pancreatectomy.	No eligible AI/ML/computational method	QuPath was used for quantitative digital pathology, but no AI/ML classifier or eligible AI-assisted tissue model was evaluated; associations were conventional.	Journal Article	eng	Langenbeck's archives of surgery	10.1007/s00423-026-04010-9	https://pubmed.ncbi.nlm.nih.gov/41801453/
42474854	Meng Z	2026	Artificial intelligence-based model for identifying anatomical and surgical structures in the videos of laparoscopic left hemicolectomy.	Wrong or non-separable population/procedure	Wrong procedure/population; not pancreatic resection	Journal Article; Multicenter Study	eng	Journal of robotic surgery	10.1007/s11701-026-03661-6	https://pubmed.ncbi.nlm.nih.gov/42474854/
42466648	Martin RC 2nd	2026	Evaluation of radiomics and radiologic signatures for diagnosis and clinical outcomes in pancreatic adenocarcinoma and pancreatic neuroendocrine: A pilot study.	Task outside focused perioperative/technical resection scope	Diagnostic/characterisation study without direct pancreatic-resection application	Journal Article	eng	Cancer biomarkers : section A of Disease markers	10.1177/18758592261455545	https://pubmed.ncbi.nlm.nih.gov/42466648/
42460832	Hampe LZ	2026	Acceptance and perceived usefulness of surgical video instructions in expert-tutored open and robotic pancreato- and hepaticojejunostomy training with 3D biotissue models.	Wrong or non-separable population/procedure	Simulation/training on 3D biotissue models; not a patient pancreatic-resection AI application.	Journal Article	eng	Minimally invasive therapy & allied technologies : MITAT : official journal of the Society for Minimally Invasive Therapy	10.1080/13645706.2026.2693302	https://pubmed.ncbi.nlm.nih.gov/42460832/
42417940	Di Martino M	2026	Perioperative corticosteroids and pancreatic surgery outcomes: a systematic review and meta-analysis combining human expertise and AI support (ChatGPT).	Non-primary publication	Non-primary publication (review/editorial/letter/guideline)	Journal Article; Review	eng	Updates in surgery	10.1007/s13304-026-02750-3	https://pubmed.ncbi.nlm.nih.gov/42417940/
42367541	Salman A	2026	Artificial Intelligence-Assisted Endoscopic Ultrasound-Guided Ablation of Pancreatic Neuroendocrine Tumors: Toward Precision Diagnosis, Risk Stratification, and Personalized Therapy.	Non-primary publication	Non-primary publication (review/editorial/letter/guideline)	Journal Article; Review	eng	Cureus	10.7759/cureus.109721	https://pubmed.ncbi.nlm.nih.gov/42367541/
42350891	Liu LY	2026	[Arterial variations and management strategies in pancreatic surgery].	Non-English publication	Non-English publication	English Abstract; Journal Article; Review	chi	Zhonghua wai ke za zhi [Chinese journal of surgery]	10.3760/cma.j.cn112139-20260109-00016	https://pubmed.ncbi.nlm.nih.gov/42350891/
42344474	Fujishima R	2026	Generative AI-Assisted Indication of Anatomical Landmarks to Enhance Safety under Intraoperative Bleeding in Laparoscopic Gastrectomy for Gastric Cancer.	Wrong or non-separable population/procedure	Wrong procedure/population; not pancreatic resection	Journal Article	eng	Annals of surgery open : perspectives of surgical history, education, and clinical approaches	10.1097/AS9.0000000000000665	https://pubmed.ncbi.nlm.nih.gov/42344474/
42305801	Yigman S	2026	The role of artificial intelligence in postoperative clinical decision-making for pancreatic cancer: a pilot study.	Task outside focused perioperative/technical resection scope	Postoperative oncological MDT/adjuvant-management decision support; not a direct pancreatic-resection/perioperative surgical application.	Journal Article	eng	Frontiers in surgery	10.3389/fsurg.2026.1802926	https://pubmed.ncbi.nlm.nih.gov/42305801/
42203661	Li BQ	2026	[Application research and evolutionary history of risk prediction models for postoperative pancreatic fistula after pancreaticoduodenectomy].	Non-English publication	Non-English publication	English Abstract; Journal Article; Review	chi	Zhonghua wai ke za zhi [Chinese journal of surgery]	10.3760/cma.j.cn112139-20250803-00390	https://pubmed.ncbi.nlm.nih.gov/42203661/
42193021	Attia A	2026	A Novel Radiomics-Integrated Panel for Preoperative Stratification of Pancreatic Neuroendocrine Tumors (PNETs).	Task outside focused perioperative/technical resection scope	Diagnostic/characterisation study without direct pancreatic-resection application	Journal Article	eng	Cancers	10.3390/cancers18101663	https://pubmed.ncbi.nlm.nih.gov/42193021/
42164679	Abdelbaki ARM	2026	Assessing postoperative pancreatic fistula risk: from subjective assessment to digital pathology-assisted quantitative precision.	Non-primary publication	Non-primary publication (review/editorial/letter/guideline)	Comment; Editorial	eng	Gland surgery	10.21037/gs-2026-1-0132	https://pubmed.ncbi.nlm.nih.gov/42164679/
42154345	Ishibayashi K	2026	Artificial intelligence-based anatomical recognition improves surgeon decision-making during robotic gastrectomy.	Wrong or non-separable population/procedure	Wrong procedure/population; not pancreatic resection	Journal Article	eng	Gastric cancer : official journal of the International Gastric Cancer Association and the Japanese Gastric Cancer Association	10.1007/s10120-026-01756-5	https://pubmed.ncbi.nlm.nih.gov/42154345/
42092273	Wang N	2026	Global Trends in Postoperative Sepsis After Pancreatoduodenectomy: A Bibliometric Analysis.	Non-primary publication	Non-primary publication (review/editorial/letter/guideline)	Journal Article; Review	eng	The Journal of surgical research	10.1016/j.jss.2026.04.010	https://pubmed.ncbi.nlm.nih.gov/42092273/
42086972	Fassari A	2026	Step-by-Step Hepatic Artery and Celiac Axis Dissection According to the Inoue Classification During Robotic Pancreaticoduodenectomy.	No eligible AI/ML/computational method	Technical operative description; no eligible AI/ML analysis demonstrated.	Journal Article; Video-Audio Media	eng	Annals of surgical oncology	10.1245/s10434-026-19742-7	https://pubmed.ncbi.nlm.nih.gov/42086972/
42085766	Gonzalez JFS	2026	Turning Ideas into Teaching Tools: How We Create Our Educational Surgical Videos.	No eligible AI/ML/computational method	Educational surgical-video methods article; no eligible AI/ML model applied to pancreatic resection.	Journal Article	eng	Journal of surgical education	10.1016/j.jsurg.2026.103967	https://pubmed.ncbi.nlm.nih.gov/42085766/
42056062	Wei YB	2026	[Clinicopathological characteristics and prognostic significance of steatotic hepatocellular carcinoma with HBV-related cirrhosis: a propensity score-matched study].	Non-English publication	Non-English publication	English Abstract; Journal Article	chi	Zhonghua wai ke za zhi [Chinese journal of surgery]	10.3760/cma.j.cn112139-20260204-00056	https://pubmed.ncbi.nlm.nih.gov/42056062/
42053950	Sahin E	2026	Multimodal AI approach combining deep learning imaging and clinical machine learning for pancreatic cancer detection.	Task outside focused perioperative/technical resection scope	Diagnostic/characterisation study without direct pancreatic-resection application	Journal Article	eng	Updates in surgery	10.1007/s13304-026-02650-6	https://pubmed.ncbi.nlm.nih.gov/42053950/
41976353	Zhang J	2026	Surgical Perspectives on Neoadjuvant Therapy in Borderline Resectable and Locally Advanced Pancreatic Cancer.	Non-primary publication	Non-primary publication (review/editorial/letter/guideline)	Journal Article; Review	eng	Cancers	10.3390/cancers18071131	https://pubmed.ncbi.nlm.nih.gov/41976353/
41868228	Ren X	2026	Precision surgery in the era of 3D visualization, AR/VR, and 3D printing: current applications and future directions.	Non-primary publication	Non-primary publication (review/editorial/letter/guideline)	Journal Article; Review	eng	Frontiers in medicine	10.3389/fmed.2026.1688748	https://pubmed.ncbi.nlm.nih.gov/41868228/
41856794	Partelli S	2026	Controversies in neuroendocrine neoplasms: An European Neuroendocrine Tumor Society position statement on the role of surgery of the primary tumour in metastatic small intestinal and pancreatic neuroendocrine tumours.	Non-primary publication	Non-primary publication (review/editorial/letter/guideline)	Consensus Statement; Journal Article; Review	eng	Journal of neuroendocrinology	10.1111/jne.70156	https://pubmed.ncbi.nlm.nih.gov/41856794/
41827668	Druzenko M	2026	Classification of Pancreatic Cancer and Normal Tissue in 2D and 3D Optical Coherence Tomography Images Using Convolutional Neural Networks: A Comparative Study.	Task outside focused perioperative/technical resection scope	Diagnostic/characterisation study without direct pancreatic-resection application	Journal Article	eng	Cancers	10.3390/cancers18050732	https://pubmed.ncbi.nlm.nih.gov/41827668/
41793556	Zhu J	2026	Bridging the innovation-evidence chasm: a critical appraisal and roadmap for robotic pancreatic cancer surgery.	Non-primary publication	Non-primary publication (review/editorial/letter/guideline)	Journal Article	eng	Journal of robotic surgery	10.1007/s11701-026-03284-x	https://pubmed.ncbi.nlm.nih.gov/41793556/
41749883	Karpes JB	2026	Reducing Complications in Pancreaticoduodenectomy.	Non-primary publication	Non-primary publication (review/editorial/letter/guideline)	Journal Article; Review	eng	Cancers	10.3390/cancers18040630	https://pubmed.ncbi.nlm.nih.gov/41749883/
41728845	Kim HS	2026	Nationwide 30-Day Surgical Outcomes in Korea: An Interim Analysis of the Korean Quality Improvement Platform in Surgery.	No eligible AI/ML/computational method	General national surgical registry; no AI model and no separable pancreatic AI analysis in the reported study.	Journal Article	eng	Journal of the American College of Surgeons	10.1097/XCS.0000000000001867	https://pubmed.ncbi.nlm.nih.gov/41728845/
41728445	Manoukian G	2026	Management of Complications After Pancreaticoduodenectomy: A Narrative Review of Pathophysiology and Treatment Strategies.	Non-primary publication	Non-primary publication (review/editorial/letter/guideline)	Journal Article; Review	eng	Cureus	10.7759/cureus.101919	https://pubmed.ncbi.nlm.nih.gov/41728445/
41674948	Wang Q	2026	Development and validation of a clinical-radiomics nomogram for the differential diagnosis of focal pancreatic solid lesions: a retrospective cohort study.	Task outside focused perioperative/technical resection scope	Diagnostic/characterisation study without direct pancreatic-resection application	Journal Article	eng	Translational cancer research	10.21037/tcr-2025-2017	https://pubmed.ncbi.nlm.nih.gov/41674948/
41549828	Ong BDC	2026	Does minimally invasive pylorus-preserving pancreaticoduodenectomy (MI-PPPD) triumph over open pylorus-preserving pancreaticoduodenectomy (O-PPPD)? A systematic review and meta-analysis.	Non-primary publication	Non-primary publication (review/editorial/letter/guideline)	Journal Article	eng	International journal of surgery (London, England)	10.1097/JS9.0000000000004734	https://pubmed.ncbi.nlm.nih.gov/41549828/
41292388	Wu XW	2026	[Artificial intelligence accelerates bridging the gap in intelligent surgery through medical-engineering-mathematical integration].	Non-English publication	Non-English publication	English Abstract; Journal Article	chi	Zhonghua wai ke za zhi [Chinese journal of surgery]	10.3760/cma.j.cn112139-20250814-00403	https://pubmed.ncbi.nlm.nih.gov/41292388/
41276443	Conticchio M	2026	Artificial intelligence in HPB surgery: a narrative review on technological advances and imperatives for ethical integration.	Non-primary publication	Non-primary publication (review/editorial/letter/guideline)	Journal Article; Review	eng	HPB : the official journal of the International Hepato Pancreato Biliary Association	10.1016/j.hpb.2025.11.002	https://pubmed.ncbi.nlm.nih.gov/41276443/
41083827	Liu H	2026	Kimura or Warshaw? Development and Validation of a Preoperative Prediction Model Based on 3D Reconstruction Technology.	No eligible AI/ML/computational method	3D reconstruction plus conventional prediction model; no AI/ML-assisted 3D method established in abstract.	Journal Article; Validation Study	eng	Annals of surgical oncology	10.1245/s10434-025-18548-3	https://pubmed.ncbi.nlm.nih.gov/41083827/
40905678	Vicente E	2026	Preoperative 3D Imaging Reconstruction Models for Predicting Infiltration of Major Vascular Structures in Patients During Pancreatic Surgery.	No eligible AI/ML/computational method	Conventional 3D reconstruction study; abstract does not establish an AI/ML-assisted reconstruction method.	Journal Article	eng	Surgical innovation	10.1177/15533506251376468	https://pubmed.ncbi.nlm.nih.gov/40905678/
40268474	Ito H	2026	Evaluation of Perioperative Cardiovascular Event Risk in Gastrointestinal Surgery - Predictive Modeling and Risk Stratification Using Machine Learning.	Wrong or non-separable population/procedure	General gastrointestinal MACE model; pancreatic/biliary surgery is only one predictor and no separable pancreatic model is reported.	Journal Article; Observational Study	eng	Circulation journal : official journal of the Japanese Circulation Society	10.1253/circj.CJ-25-0032	https://pubmed.ncbi.nlm.nih.gov/40268474/
39676639	Ahmad MU	2026	Biochemical, Radiographic, or Pathologic Response to Neoadjuvant Chemotherapy in Resected Pancreatic Cancer: Which Is Best?	Task outside focused perioperative/technical resection scope	Oncological prognosis/recurrence outcome without direct perioperative surgical application	Journal Article; Research Support, Non-U.S. Gov't	eng	Annals of surgery	10.1097/SLA.0000000000006609	https://pubmed.ncbi.nlm.nih.gov/39676639/
41469859	Yang C	2025	Constructing a machine learning model for predicting early postoperative recurrence of pancreatic head cancer based on a novel inflammatory factor composite index.	Task outside focused perioperative/technical resection scope	Oncological prognosis/recurrence outcome without direct perioperative surgical application	Journal Article	eng	BMC cancer	10.1186/s12885-025-15493-y	https://pubmed.ncbi.nlm.nih.gov/41469859/

PMID	Study	Year	Title	Detailed-assessment exclusion category	Specific exclusion reason	Publication type	Language	Journal	DOI	PubMed URL
41388696	Cai Z	2025	Deep learning to predict extrapancreatic perineural invasion at CT images.	Task outside focused perioperative/technical resection scope	Imaging prediction of perineural invasion; diagnostic/staging task without a direct resection application.	Journal Article; Research Support, Non-U.S. Gov't	eng	Annals of medicine	10.1080/07853890.2025.2568116	https://pubmed.ncbi.nlm.nih.gov/41388696/
41375676	Caringi S	2025	Robotic Pancreaticoduodenectomy: Current Evidence and Future Perspectives.	Non-primary publication	Non-primary publication (review/editorial/letter/guideline)	Journal Article; Review	eng	Journal of clinical medicine	10.3390/jcm14238372	https://pubmed.ncbi.nlm.nih.gov/41375676/
41357630	Martli HF	2025	Clinicopathological features of patients undergoing surgery for pancreatic cancer with very early postoperative recurrence.	Task outside focused perioperative/technical resection scope	Oncological prognosis/recurrence outcome without direct perioperative surgical application	Journal Article	eng	World journal of gastrointestinal surgery	10.4240/wjgs.v17.i11.111202	https://pubmed.ncbi.nlm.nih.gov/41357630/
41010705	Efstathiou A	2025	Artificial Intelligence and Digital Tools Across the Hepato-Pancreato-Biliary Surgical Pathway: A Systematic Review.	Non-primary publication	Non-primary publication (review/editorial/letter/guideline)	Journal Article; Review	eng	Journal of clinical medicine	10.3390/jcm14186501	https://pubmed.ncbi.nlm.nih.gov/41010705/
41005002	Nakao E	2025	Effectiveness of artificial intelligence-based visualization for surgical anatomy education: A cluster quasirandomized controlled trial.	Wrong or non-separable population/procedure	AI surgical anatomy education study based on gastrectomy videos; not pancreatic resection.	Journal Article; Randomized Controlled Trial	eng	Surgery	10.1016/j.surg.2025.109723	https://pubmed.ncbi.nlm.nih.gov/41005002/
41003776	Ikeda J	2025	Assessment of AI-driven anatomical recognition in robotic gastrectomy: A multicenter retrospective analysis.	Wrong or non-separable population/procedure	Wrong procedure/population; not pancreatic resection	Journal Article; Multicenter Study	eng	Surgical endoscopy	10.1007/s00464-025-12248-5	https://pubmed.ncbi.nlm.nih.gov/41003776/
40944744	Okumura S	2025	Artificial intelligence-supported system of surgical anatomy recognition may facilitate the understanding of gastrointestinal surgery for medical students.	Wrong or non-separable population/procedure	AI anatomy education based on distal gastrectomy videos; pancreas is an adjacent structure, not pancreatic resection.	Journal Article	eng	Surgical endoscopy	10.1007/s00464-025-12205-2	https://pubmed.ncbi.nlm.nih.gov/40944744/
40921854	Ri M	2025	Time-sequential prediction of postoperative complications after gastric cancer surgery using machine learning: a multicenter cohort study.	Wrong or non-separable population/procedure	Wrong procedure/population; not pancreatic resection	Journal Article; Multicenter Study	eng	Gastric cancer : official journal of the International Gastric Cancer Association and the Japanese Gastric Cancer Association	10.1007/s10120-025-01658-y	https://pubmed.ncbi.nlm.nih.gov/40921854/
40899628	Grafenhorst E	2025	DNA methylation profile to aid in the diagnosis of pancreatic ductal adenocarcinoma and its role in disease progression.	Non-primary publication	Non-primary publication (review/editorial/letter/guideline)	Journal Article; Review	eng	Epigenomics	10.1080/17501911.2025.2554571	https://pubmed.ncbi.nlm.nih.gov/40899628/
40861574	Mohammed C	2025	Evolving Surgical Approaches in the Management of Pancreatic Masses: From Open Resection to Minimally Invasive and Robotic Techniques.	Non-primary publication	Non-primary publication (review/editorial/letter/guideline)	Journal Article; Review	eng	Cureus	10.7759/cureus.88607	https://pubmed.ncbi.nlm.nih.gov/40861574/
40831131	Ping L	2025	[Exploration of the application of artificial intelligence assisted bleeding point recognition in laparoscopic pancreatic surgery].	Non-English publication	Non-English publication	English Abstract; Journal Article	chi	Zhonghua wai ke za zhi [Chinese journal of surgery]	10.3760/cma.j.cn112139-20250424-00218	https://pubmed.ncbi.nlm.nih.gov/40831131/
40666589	Chen G	2025	The Role of Large Language Models (LLMs) in Hepato-Pancreato-Biliary Surgery: Opportunities and Challenges.	Non-primary publication	Non-primary publication (review/editorial/letter/guideline)	Editorial	eng	Cureus	10.7759/cureus.85979	https://pubmed.ncbi.nlm.nih.gov/40666589/
40640275	Rastkhiz A	2025	Preoperative arterial and venous CT radiomics for survival prediction after pylorus preserving pancreatoduodenectomy in pancreatic head cancer.	Task outside focused perioperative/technical resection scope	Oncological prognosis/recurrence outcome without direct perioperative surgical application	Journal Article	eng	Scientific reports	10.1038/s41598-025-09969-x	https://pubmed.ncbi.nlm.nih.gov/40640275/
40556394	Guo XJ	2025	[Current situation and development trends of minimally invasive surgical procedures in the treatment of pancreatic tumors].	Non-English publication	Non-English publication	English Abstract; Journal Article; Review	chi	Zhonghua wai ke za zhi [Chinese journal of surgery]	10.3760/cma.j.cn112139-20250224-00090	https://pubmed.ncbi.nlm.nih.gov/40556394/
40519766	Fahlbusch T	2025	Use of Histomorphological Features for the Prediction of Grade C Fistula after Whipple Procedure: A Difficult Task.	No eligible AI/ML/computational method	Conventional histomorphological fistula-risk study; no eligible AI/ML/digital-pathology model.	Journal Article	eng	Visceral medicine	10.1159/000543436	https://pubmed.ncbi.nlm.nih.gov/40519766/
40296678	Qi Y	2025	High-Precision Intelligent Diagnosis of Pancreatic Cancer: Flowing Diffuseness from Single to Whole.	Task outside focused perioperative/technical resection scope	Diagnostic/characterisation study without direct pancreatic-resection application	Journal Article; Research Support, Non-U.S. Gov't	eng	Analytical chemistry	10.1021/acs.analchem.5c00465	https://pubmed.ncbi.nlm.nih.gov/40296678/
39985003	Zhang TQ	2025	De novo familial adenomatous polyposis with germline double heterozygosity of APC/BRCA2: a case report and literature review.	Non-primary publication	Non-primary publication (review/editorial/letter/guideline)	Journal Article	eng	Hereditary cancer in clinical practice	10.1186/s13053-025-00306-x	https://pubmed.ncbi.nlm.nih.gov/39985003/
39920789	Li T	2025	Evaluating the surgical and oncological outcomes of hepatic artery variations in minimally invasive pancreaticoduodenectomy: insights from 2023 data at a high-volume pancreatic center.	No eligible AI/ML/computational method	Anatomical/outcome study of hepatic artery variation; no eligible AI/ML method.	Journal Article	eng	World journal of surgical oncology	10.1186/s12957-025-03704-6	https://pubmed.ncbi.nlm.nih.gov/39920789/
39915344	Alhulaili ZM	2025	Risk stratification of postoperative pancreatic fistula and other complications following pancreatoduodenectomy. How far are we? A scoping review.	Non-primary publication	Non-primary publication (review/editorial/letter/guideline)	Journal Article; Scoping Review	eng	Langenbeck's archives of surgery	10.1007/s00423-024-03581-9	https://pubmed.ncbi.nlm.nih.gov/39915344/
39869381	Laga Boul-Atarass I	2025	Virtual 3D models, augmented reality systems and virtual laparoscopic simulations in complicated pancreatic surgeries: state of art, future perspectives, and challenges.	Non-primary publication	Non-primary publication (review/editorial/letter/guideline)	Journal Article; Review	eng	International journal of surgery (London, England)	10.1097/JS9.0000000000002231	https://pubmed.ncbi.nlm.nih.gov/39869381/
39616771	Nakamura T	2025	The potential of AI-assisted gastrectomy with dual highlighting of pancreas and connective tissue.	Wrong or non-separable population/procedure	Wrong procedure/population; not pancreatic resection	Journal Article; Video-Audio Media	eng	Surgical oncology	10.1016/j.suronc.2024.102171	https://pubmed.ncbi.nlm.nih.gov/39616771/
39513271	Jia Z	2025	Identification and Categorization of Technical Errors and Hazard Zones of Robotic Versus Laparoscopic Total Gastrectomy for Gastric Cancer: A Single-center Prospective Randomized Controlled Study.	Wrong or non-separable population/procedure	Wrong procedure/population; not pancreatic resection	Comparative Study; Journal Article; Randomized Controlled Trial; Research Support, Non-U.S. Gov't	eng	Annals of surgery	10.1097/SLA.0000000000006585	https://pubmed.ncbi.nlm.nih.gov/39513271/
39228175	Ducas A	2025	The role of artificial intelligence in pancreatic surgery: Current and future perspectives.	Non-primary publication	Non-primary publication (review/editorial/letter/guideline)	Editorial	eng	Annals of hepato-biliary-pancreatic surgery	10.14701/ahbps.24-130	https://pubmed.ncbi.nlm.nih.gov/39228175/
39296003	Sun Y	2024	Non-invasive diagnosis of pancreatic steatosis with ultrasound images using deep learning network.	Task outside focused perioperative/technical resection scope	Diagnostic/characterisation study without direct pancreatic-resection application	Journal Article	eng	Heliyon	10.1016/j.heliyon.2024.e37580	https://pubmed.ncbi.nlm.nih.gov/39296003/
39200119	Yang J	2024	Remnant Pancreas Volume Affects New-Onset Impaired Glucose Homeostasis Secondary to Pancreatic Cancer.	No eligible AI/ML/computational method	Conventional remnant-volume association study; no eligible AI/ML/radiomics method.	Journal Article	eng	Biomedicines	10.3390/biomedicines12081653	https://pubmed.ncbi.nlm.nih.gov/39200119/
39183018	Cheng SB	2024	[Construction and verification of pancreatic fistula risk prediction model after pancreaticoduodenectomy based on ensemble machine learning].	Non-English publication	Non-English publication	English Abstract; Journal Article	chi	Zhonghua wai ke za zhi [Chinese journal of surgery]	10.3760/cma.j.cn112139-20240411-00180	https://pubmed.ncbi.nlm.nih.gov/39183018/
39133331	Aoyama Y	2024	Correction: Artificial intelligence for surgical safety during laparoscopic gastrectomy for gastric cancer: Indication of anatomical landmarks related to postoperative pancreatic fistula using deep learning.	Non-primary publication	Non-primary publication (review/editorial/letter/guideline)	Published Erratum	eng	Surgical endoscopy	10.1007/s00464-024-11160-8	https://pubmed.ncbi.nlm.nih.gov/39133331/
39120842	Yee EJ	2024	Dynamic Anthropometrics in Pancreatic Cancer: Associations Between Body Composition Changes During Neoadjuvant Therapy and Survival Outcomes After Resection.	Task outside focused perioperative/technical resection scope	Oncological prognosis/recurrence outcome without direct perioperative surgical application	Journal Article	eng	Annals of surgical oncology	10.1245/s10434-024-15975-6	https://pubmed.ncbi.nlm.nih.gov/39120842/
39093411	Aoyama Y	2024	Artificial intelligence for surgical safety during laparoscopic gastrectomy for gastric cancer: Indication of anatomical landmarks related to postoperative pancreatic fistula using deep learning.	Wrong or non-separable population/procedure	Wrong procedure/population; not pancreatic resection	Journal Article; Research Support, Non-U.S. Gov't	eng	Surgical endoscopy	10.1007/s00464-024-11117-x	https://pubmed.ncbi.nlm.nih.gov/39093411/
38968550	Yoo J	2024	Prognostic Significance of 18 F-FDG PET/CT Radiomics in Patients With Resectable Pancreatic Ductal Adenocarcinoma Undergoing Curative Surgery.	Task outside focused perioperative/technical resection scope	Oncological prognosis/recurrence outcome without direct perioperative surgical application	Journal Article	eng	Clinical nuclear medicine	10.1097/RLU.0000000000005363	https://pubmed.ncbi.nlm.nih.gov/38968550/
38778686	Zhang KC	2024	[Computer-vision-based artificial intelligence for detection and recognition of instruments and organs during radical laparoscopic gastrectomy for gastric cancer: a multicenter study].	Non-English publication	Non-English publication	English Abstract; Journal Article; Multicenter Study	chi	Zhonghua wei chang wai ke za zhi = Chinese journal of gastrointestinal surgery	10.3760/cma.j.cn441530-20240125-00041	https://pubmed.ncbi.nlm.nih.gov/38778686/
38596504	Yang F	2024	Optimizing prediction models for pancreatic fistula after pancreatectomy: Current status and future perspectives.	Non-primary publication	Non-primary publication (review/editorial/letter/guideline)	Journal Article; Review	eng	World journal of gastroenterology	10.3748/wjg.v30.i10.1329	https://pubmed.ncbi.nlm.nih.gov/38596504/
38573374	Nakamura T	2024	Precise highlighting of the pancreas by semantic segmentation during robot-assisted gastrectomy: visual assistance with artificial intelligence for surgeons.	Wrong or non-separable population/procedure	Wrong procedure/population; not pancreatic resection	Journal Article; Research Support, Non-U.S. Gov't	eng	Gastric cancer : official journal of the International Gastric Cancer Association and the Japanese Gastric Cancer Association	10.1007/s10120-024-01495-5	https://pubmed.ncbi.nlm.nih.gov/38573374/
38568375	Ashraf Ganjouei A	2024	Reply to: Letter to Editor Regarding Article "A Machine Learning Approach to Predict Postoperative Pancreatic Fistula after Pancreaticoduodenectomy Using Only Preoperatively Known Data" by Yang, Yanfei et al.	Non-primary publication	Non-primary publication (review/editorial/letter/guideline)	Letter	eng	Annals of surgical oncology	10.1245/s10434-024-15240-w	https://pubmed.ncbi.nlm.nih.gov/38568375/
38557910	Hu H	2024	Letter to Editor Regarding Article "A Machine Learning Approach to Predict Postoperative Pancreatic Fistula After Pancreaticoduodenectomy Using Only Preoperatively Known Data".	Non-primary publication	Non-primary publication (review/editorial/letter/guideline)	Letter	eng	Annals of surgical oncology	10.1245/s10434-024-15237-5	https://pubmed.ncbi.nlm.nih.gov/38557910/

PMID	Study	Year	Title	Detailed-assessment exclusion category	Specific exclusion reason	Publication type	Language	Journal	DOI	PubMed URL
38416748	Uraoka M	2024	Novel embryological classifications of hepatic arteries based on the relationship between aberrant right hepatic arteries and the middle hepatic artery: A retrospective study of contrast-enhanced computed tomography images.	No eligible AI/ML/computational method	Anatomical CT classification study; no direct pancreatic-resection AI task.	Journal Article	eng	PloS one	10.1371/journal.pone.0299263	https://pubmed.ncbi.nlm.nih.gov/38416748/
38385553	Koziol-Bohatkiewicz P	2024	Fast cancer imaging in pancreatic biopsies using infrared imaging.	Task outside focused perioperative/technical resection scope	Cancer diagnosis in pancreatic biopsy specimens; not a resection-specific surgical application.	Journal Article	eng	The Analyst	10.1039/d3an01555f	https://pubmed.ncbi.nlm.nih.gov/38385553/
38322062	Nayak K	2024	Surgical Perspectives of Open vs. Laparoscopic Approaches to Lateral Pancreaticojejunostomy: A Comprehensive Review.	Non-primary publication	Non-primary publication (review/editorial/letter/guideline)	Journal Article; Review	eng	Cureus	10.7759/cureus.51769	https://pubmed.ncbi.nlm.nih.gov/38322062/
38278723	Kitaguchi D	2024	Free-Viewpoint Video in Open Surgery: Development of Surgical Arena 360.	Wrong or non-separable population/procedure	General open-surgery free-viewpoint video technology; no pancreas-specific resection analysis.	Journal Article; Research Support, Non-U.S. Gov't; Video-Audio Media	eng	Journal of surgical education	10.1016/j.jsurg.2023.12.003	https://pubmed.ncbi.nlm.nih.gov/38278723/
38238193	Ingwersen EW	2024	Radiomics for the prediction of a postoperative pancreatic fistula following a pancreatoduodenectomy: A systematic review and radiomic score quality assessment.	Non-primary publication	Non-primary publication (review/editorial/letter/guideline)	Journal Article; Systematic Review	eng	Pancreatology : official journal of the International Association of Pancreatology (IAP) ... [et al.]	10.1016/j.pan.2023.12.007	https://pubmed.ncbi.nlm.nih.gov/38238193/
38194643	Kiemen AL	2024	Magnetic Resonance Imaging-Based Assessment of Pancreatic Fat Strongly Correlates With Histology-Based Assessment of Pancreas Composition.	No eligible AI/ML/computational method	MRI-fat versus histology correlation study; no eligible AI/ML/radiomics prediction task.	Journal Article; Research Support, N.I.H., Extramural; Research Support, Non-U.S. Gov't	eng	Pancreas	10.1097/MPA.0000000000002288	https://pubmed.ncbi.nlm.nih.gov/38194643/
38191489	Lee JH	2024	Preoperative prediction of early recurrence in resectable pancreatic cancer integrating clinical, radiologic, and CT radiomics features.	Task outside focused perioperative/technical resection scope	Oncological prognosis/recurrence outcome without direct perioperative surgical application	Journal Article	eng	Cancer imaging : the official publication of the International Cancer Imaging Society	10.1186/s40644-024-00653-3	https://pubmed.ncbi.nlm.nih.gov/38191489/
38106231	Forjaz A	2024	Three-dimensional assessments are necessary to determine the true, spatially-resolved composition of tissues.	Wrong or non-separable population/procedure	General 3D tissue-composition methodology; not a pancreatic-resection AI application.	Journal Article; Preprint	eng	bioRxiv : the preprint server for biology	10.1101/2023.12.04.569986	https://pubmed.ncbi.nlm.nih.gov/38106231/
38085802	Chu LC	2024	Pancreatic ductal adenocarcinoma staging: a narrative review of radiologic techniques and advances.	Non-primary publication	Non-primary publication (review/editorial/letter/guideline)	Journal Article; Review	eng	International journal of surgery (London, England)	10.1097/JS9.0000000000000899	https://pubmed.ncbi.nlm.nih.gov/38085802/
38040594	Muaddi H	2024	Persistent challenges in pancreatic surgery: Postoperative pancreatic fistula prediction in the machine learning era-Response to: Machine learning versus logistic regression for the prediction of complications after pancreaticoduodenectomy.	Non-primary publication	Non-primary publication (review/editorial/letter/guideline)	Comment; Letter	eng	Surgery	10.1016/j.surg.2023.10.036	https://pubmed.ncbi.nlm.nih.gov/38040594/
38038811	Komatsu M	2024	Automatic surgical phase recognition-based skill assessment in laparoscopic distal gastrectomy using multicenter videos.	Wrong or non-separable population/procedure	Wrong procedure/population; not pancreatic resection	Journal Article; Multicenter Study	eng	Gastric cancer : official journal of the International Gastric Cancer Association and the Japanese Gastric Cancer Association	10.1007/s10120-023-01450-w	https://pubmed.ncbi.nlm.nih.gov/38038811/
37914911	Zhai Y	2024	Artificial intelligence for automatic surgical phase recognition of laparoscopic gastrectomy in gastric cancer.	Wrong or non-separable population/procedure	Wrong procedure/population; not pancreatic resection	Journal Article; Video-Audio Media	eng	International journal of computer assisted radiology and surgery	10.1007/s11548-023-03027-5	https://pubmed.ncbi.nlm.nih.gov/37914911/
37450702	Abu Hilal M	2024	The Brescia Internationally Validated European Guidelines on Minimally Invasive Pancreatic Surgery (EGUMIPS).	Non-primary publication	Non-primary publication (review/editorial/letter/guideline)	Journal Article	eng	Annals of surgery	10.1097/SLA.0000000000006006	https://pubmed.ncbi.nlm.nih.gov/37450702/
38249287	Mejia A	2023	Albumin-Bilirubin (ALBI) Score and Systemic Immune-Inflammation Indexes Used As Pretreatment Outcome Predictors in Patients With Pancreatic Ductal Adenocarcinoma Undergoing Robotic or Open Whipple Procedures: A Logistic Regression Analysis.	No eligible AI/ML/computational method	Conventional logistic-regression prognostic analysis; no eligible AI/ML application.	Journal Article	eng	Cureus	10.7759/cureus.50949	https://pubmed.ncbi.nlm.nih.gov/38249287/
38068432	Pacella G	2023	Pancreatic Ductal Adenocarcinoma: Update of CT-Based Radiomics Applications in the Pre-Surgical Prediction of the Risk of Post-Operative Fistula, Resectability Status and Prognosis.	Non-primary publication	Non-primary publication (review/editorial/letter/guideline)	Journal Article; Review	eng	Journal of clinical medicine	10.3390/jcm12237380	https://pubmed.ncbi.nlm.nih.gov/38068432/
37731637	Cen C	2023	Clinical-radiomics nomogram using contrast-enhanced CT to predict histological grade and survival in pancreatic ductal adenocarcinoma.	Task outside focused perioperative/technical resection scope	Oncological prognosis/recurrence outcome without direct perioperative surgical application	Journal Article	eng	Frontiers in oncology	10.3389/fonc.2023.1218128	https://pubmed.ncbi.nlm.nih.gov/37731637/
37653993	Ge YP	2023	[A single-center retrospective study of percutaneous drainage clinical characteristics of grade B and C postoperative pancreatic fistula and determination of the optimal intervention time].	Non-English publication	Non-English publication	English Abstract; Journal Article	chi	Zhonghua wai ke za zhi [Chinese journal of surgery]	10.3760/cma.j.cn112139-20230319-00113	https://pubmed.ncbi.nlm.nih.gov/37653993/
37610488	Ashraf Ganjouei A	2023	ASO Author Reflections: Machine Learning-Based Preoperative Prediction of Pancreatic Fistula after Pancreaticoduodenectomy.	Non-primary publication	Non-primary publication (review/editorial/letter/guideline)	Comment; Journal Article	eng	Annals of surgical oncology	10.1245/s10434-023-14152-5	https://pubmed.ncbi.nlm.nih.gov/37610488/
37526099	Kolbinger FR	2023	Anatomy segmentation in laparoscopic surgery: comparison of machine learning and human expertise - an experimental study.	Wrong or non-separable population/procedure	General laparoscopic anatomy-segmentation experiment; no pancreatic-resection cohort.	Journal Article	eng	International journal of surgery (London, England)	10.1097/JS9.0000000000000595	https://pubmed.ncbi.nlm.nih.gov/37526099/
37461042	Nguyen TK	2023	Pancreaticoduodenectomy combined gastroduodenal collateral reconstruction and preservation due to median arcuate ligament syndrome: technical notes with two surgical cases report (with video).	No eligible AI/ML/computational method	Technical case report/video; no eligible AI/ML analysis.	Case Reports; Journal Article; Video-Audio Media	eng	World journal of surgical oncology	10.1186/s12957-023-03096-5	https://pubmed.ncbi.nlm.nih.gov/37461042/
37383385	Taha A	2023	Machine learning in pancreas surgery, what is new? literature review.	Non-primary publication	Non-primary publication (review/editorial/letter/guideline)	Journal Article; Review	eng	Frontiers in surgery	10.3389/fsurg.2023.1142585	https://pubmed.ncbi.nlm.nih.gov/37383385/
36635312	Carstens M	2023	The Dresden Surgical Anatomy Dataset for Abdominal Organ Segmentation in Surgical Data Science.	Wrong or non-separable population/procedure	Surgical anatomy dataset derived from non-pancreatic operations; no pancreatic-resection video cohort.	Dataset; Journal Article	eng	Scientific data	10.1038/s41597-022-01719-2	https://pubmed.ncbi.nlm.nih.gov/36635312/
36611070	Kiritani S	2023	New Diagnostic Modality Combining Mass Spectrometry and Machine Learning for the Discrimination of Malignant Intraductal Papillary Mucinous Neoplasms.	Task outside focused perioperative/technical resection scope	ML diagnosis of malignant IPMN; diagnostic classification without direct resection application.	Journal Article	eng	Annals of surgical oncology	10.1245/s10434-022-13012-y	https://pubmed.ncbi.nlm.nih.gov/36611070/
35781511	Yao J	2023	Deep Learning for Fully Automated Prediction of Overall Survival in Patients Undergoing Resection for Pancreatic Cancer: A Retrospective Multicenter Study.	Wrong or non-separable population/procedure	Deep-learning overall-survival prognostication after resection; oncological prognosis/adjuvant selection rather than direct perioperative surgical application.	Journal Article; Multicenter Study	eng	Annals of surgery	10.1097/SLA.0000000000005465	https://pubmed.ncbi.nlm.nih.gov/35781511/
36588487	Pavlidis ET	2022	Modern aspects of the management of pancreatic intraductal papillary mucinous neoplasms: a narrative review.	Non-primary publication	Non-primary publication (review/editorial/letter/guideline)	Journal Article; Review	eng	Romanian journal of morphology and embryology = Revue roumaine de morphologie et embryologie	10.47162/RJME.63.3.03	https://pubmed.ncbi.nlm.nih.gov/36588487/
36568148	Chang J	2022	Machine-learning based investigation of prognostic indicators for oncological outcome of pancreatic ductal adenocarcinoma.	Task outside focused perioperative/technical resection scope	Oncological prognosis/recurrence outcome without direct perioperative surgical application	Journal Article	eng	Frontiers in oncology	10.3389/fonc.2022.895515	https://pubmed.ncbi.nlm.nih.gov/36568148/
35839563	Nagai K	2022	Pancreatoduodenectomy with portal vein resection for pancreatic body cancer in a patient with situs inversus totalis.	No eligible AI/ML/computational method	Pancreatoduodenectomy case report; no eligible AI/ML method.	Case Reports; Journal Article; Video-Audio Media	eng	Surgical oncology	10.1016/j.suronc.2022.101815	https://pubmed.ncbi.nlm.nih.gov/35839563/
35794703	Kim H	2022	Comparison of survival prediction models for pancreatic cancer: Cox model versus machine learning models.	Task outside focused perioperative/technical resection scope	Oncological prognosis/recurrence outcome without direct perioperative surgical application	Journal Article	eng	Genomics & informatics	10.5808/gi.22036	https://pubmed.ncbi.nlm.nih.gov/35794703/
35726190	Taherian M	2022	Critical issues in pathologic evaluation of pancreatic ductal adenocarcinoma resected after neoadjuvant treatment: a narrative review.	Non-primary publication	Non-primary publication (review/editorial/letter/guideline)	Journal Article; Review	eng	Chinese clinical oncology	10.21037/cco-21-175	https://pubmed.ncbi.nlm.nih.gov/35726190/
35667953	Tung CL	2022	Identifying pathological slices of gastric cancer via deep learning.	Wrong or non-separable population/procedure	Gastric-cancer pathology deep learning; wrong disease/procedure.	Journal Article	eng	Journal of the Formosan Medical Association = Taiwan yi zhi	10.1016/j.jfma.2022.05.004	https://pubmed.ncbi.nlm.nih.gov/35667953/
35378551	Litkevych S	2022	[Robotic-assisted Enucleation of a Cystic Neoplasm in the Head of the Pancreas].	Non-English publication	Non-English publication	Case Reports; Journal Article	ger	Zentralblatt fur Chirurgie	10.1055/a-1768-1123	https://pubmed.ncbi.nlm.nih.gov/35378551/
35317424	Ardeshna DR	2022	Recent advances in the diagnostic evaluation of pancreatic cystic lesions.	Non-primary publication	Non-primary publication (review/editorial/letter/guideline)	Journal Article; Review	eng	World journal of gastroenterology	10.3748/wjg.v28.i6.624	https://pubmed.ncbi.nlm.nih.gov/35317424/
35237939	Schlanger D	2022	The role of artificial intelligence in pancreatic surgery: a systematic review.	Non-primary publication	Non-primary publication (review/editorial/letter/guideline)	Journal Article; Systematic Review	eng	Updates in surgery	10.1007/s13304-022-01255-z	https://pubmed.ncbi.nlm.nih.gov/35237939/
35230581	Hayashi K	2022	Prediction of Recurrence Pattern of Pancreatic Cancer Post-Pancreatic Surgery Using Histology-Based Supervised Machine Learning Algorithms: A Single-Center Retrospective Study.	Task outside focused perioperative/technical resection scope	Oncological prognosis/recurrence outcome without direct perioperative surgical application	Journal Article	eng	Annals of surgical oncology	10.1245/s10434-022-11471-x	https://pubmed.ncbi.nlm.nih.gov/35230581/
34889852	Wang H	2022	Pathologic Examination of Pancreatic Specimens Resected for Treated Pancreatic Ductal Adenocarcinoma: Recommendations From the Pancreatobiliary Pathology Society.	Non-primary publication	Pathology recommendations/guidance rather than eligible primary AI study.	Journal Article; Research Support, N.I.H., Extramural	eng	The American journal of surgical pathology	10.1097/PAS.0000000000001853	https://pubmed.ncbi.nlm.nih.gov/34889852/
34368928	Zhao G	2022	The standardized technique and surgical video of robotic pancreaticoduodenectomy at the Chinese PLA General Hospital.	No eligible AI/ML/computational method	Educational/technical pancreaticoduodenectomy video; no AI/ML analysis.	Journal Article; Video-Audio Media	eng	Updates in surgery	10.1007/s13304-021-01149-6	https://pubmed.ncbi.nlm.nih.gov/34368928/

PMID	Study	Year	Title	Detailed-assessment exclusion category	Specific exclusion reason	Publication type	Language	Journal	DOI	PubMed URL
34738844	Baig Z	2021	Prognosticating Outcome in Pancreatic Head Cancer With the use of a Machine Learning Algorithm.	Task outside focused perioperative/technical resection scope	Oncological prognosis/recurrence outcome without direct perioperative surgical application	Journal Article; Research Support, Non-U.S. Gov't	eng	Technology in cancer research & treatment	10.1177/15330338211050767	https://pubmed.ncbi.nlm.nih.gov/34738844/
34654961	Zhou Y	2021	A novel anastomosis technique facilitates pancreaticojejunostomy in total laparoscopic pancreaticoduodenectomy (with video).	No eligible AI/ML/computational method	Pancreaticojejunostomy technique paper with a surgical video but no AI/ML/video-analysis model.	Journal Article; Video-Audio Media	eng	Langenbeck's archives of surgery	10.1007/s00423-021-02347-x	https://pubmed.ncbi.nlm.nih.gov/34654961/
34638421	Palumbo D	2021	Prediction of Early Distant Recurrence in Upfront Resectable Pancreatic Adenocarcinoma: A Multidisciplinary, Machine Learning-Based Approach.	Task outside focused perioperative/technical resection scope	Oncological prognosis/recurrence outcome without direct perioperative surgical application	Journal Article	eng	Cancers	10.3390/cancers13194938	https://pubmed.ncbi.nlm.nih.gov/34638421/
34619904	Fang CH	2021	[Application of digital medical technology in hepatopancreatobiliary surgery:20 years' retrospective review and prospect].	Non-English publication	Non-English publication	Journal Article	chi	Zhonghua wai ke za zhi [Chinese journal of surgery]	10.3760/cma.j.cn112139-20210405-00159	https://pubmed.ncbi.nlm.nih.gov/34619904/
34412188	Wang XJ	2021	[Anatomical observation of the right retroperitoneal fascia and its clinical significance in complete mesocolic excision for right colon cancer].	Non-English publication	Non-English publication	Journal Article	chi	Zhonghua wei chang wai ke za zhi = Chinese journal of gastrointestinal surgery	10.3760/cma.j.cn.441530-20210121-00034	https://pubmed.ncbi.nlm.nih.gov/34412188/
34357472	Schlitter AM	2021	[Standardized diagnosis of pancreatic head carcinoma].	Non-English publication	Non-English publication	Journal Article; Review	ger	Der Pathologe	10.1007/s00292-021-00971-4	https://pubmed.ncbi.nlm.nih.gov/34357472/
34055620	Wei M	2021	A Novel Validated Recurrence Stratification System Based on (18)F-FDG PET/CT Radiomics to Guide Surveillance After Resection of Pancreatic Cancer.	Task outside focused perioperative/technical resection scope	Oncological prognosis/recurrence outcome without direct perioperative surgical application	Journal Article	eng	Frontiers in oncology	10.3389/fonc.2021.650266	https://pubmed.ncbi.nlm.nih.gov/34055620/
33552391	Bari H	2021	Role of artificial intelligence in hepatobiliary and pancreatic surgery.	Non-primary publication	Non-primary publication (review/editorial/letter/guideline)	Journal Article; Review	eng	World journal of gastrointestinal surgery	10.4240/wjgs.v13.i1.7	https://pubmed.ncbi.nlm.nih.gov/33552391/
33500172	Liu H	2021	Application of three-dimensional reconstruction with a Hisense computer-assisted system in upper pancreatic lymph node dissection during laparoscopic-assisted radical gastrectomy.	Wrong or non-separable population/procedure	Wrong procedure/population; not pancreatic resection	Journal Article	eng	Asian journal of surgery	10.1016/j.asjsur.2020.12.034	https://pubmed.ncbi.nlm.nih.gov/33500172/
33381979	Watson MD	2021	Pure and Hybrid Deep Learning Models can Predict Pathologic Tumor Response to Neoadjuvant Therapy in Pancreatic Adenocarcinoma: A Pilot Study.	Task outside focused perioperative/technical resection scope	Oncological prognosis/recurrence outcome without direct perioperative surgical application	Journal Article	eng	The American surgeon	10.1177/0003134820982557	https://pubmed.ncbi.nlm.nih.gov/33381979/
33053264	Zhou HT	2021	Quantitative analysis of three-dimensional reconstruction data to guide the selection of methods for laparoscopic distal pancreatectomy.	No eligible AI/ML/computational method	Conventional 3D reconstruction used for DP approach selection; no AI/ML component established.	Journal Article	eng	Journal of hepato-biliary-pancreatic sciences	10.1002/jhbp.849	https://pubmed.ncbi.nlm.nih.gov/33053264/
33217652	Gadelha Bezerra Silva B	2020	Management of gastric ectopic pancreas identified in preoperative evaluation of bariatric surgery - A case report.	Wrong or non-separable population/procedure	Wrong procedure/population; not pancreatic resection	Case Reports; Journal Article	eng	International journal of surgery case reports	10.1016/j.ijscr.2020.10.104	https://pubmed.ncbi.nlm.nih.gov/33217652/
32891320	Paik KY Prof	2020	Lateral approach toward hepatoduodenal ligament during laparoscopic radical cholecystectomy for Gallbladder cancer.	Wrong or non-separable population/procedure	Wrong procedure/population; not pancreatic resection	Case Reports; Journal Article	eng	Surgical oncology	10.1016/j.suronc.2020.04.009	https://pubmed.ncbi.nlm.nih.gov/32891320/
32613028	Harrington KA	2020	Multimodal radiomics and cyst fluid inflammatory markers model to predict preoperative risk in intraductal papillary mucinous neoplasms.	Task outside focused perioperative/technical resection scope	Preoperative IPMN malignancy-risk model; diagnostic classification rather than direct resection application.	Journal Article	eng	Journal of medical imaging (Bellingham, Wash.)	10.1117/1.JMI.7.3.031507	https://pubmed.ncbi.nlm.nih.gov/32613028/
32284918	Latif E	2020	Idiopathic Retroperitoneal Non-pancreatic Pseudocyst in an Adult Male: Radiological Images and Surgical Video of Laparoscopic Excision.	Wrong or non-separable population/procedure	Non-pancreatic pseudocyst surgery; wrong population.	Case Reports; Journal Article	eng	Cureus	10.7759/cureus.7243	https://pubmed.ncbi.nlm.nih.gov/32284918/
31993802	Oikawa R	2020	Arterial communication around the pancreatic tail enabled division of the gastroduodenal artery during pancreaticoduodenectomy in patient with complete celiac artery occlusion: a case report.	No eligible AI/ML/computational method	Technical case report; no eligible AI/ML method.	Journal Article	eng	Surgical case reports	10.1186/s40792-020-0787-2	https://pubmed.ncbi.nlm.nih.gov/31993802/
31953732	Giulianotti PC	2020	Educational step-by-step surgical video about operative technique in robotic pancreaticoduodenectomy (RPD) at University of Illinois at Chicago (UIC): 17 steps standardized technique-Lessons learned since the first worldwide RPD performed in the year 2001.	No eligible AI/ML/computational method	Educational operative video; no eligible AI/ML analysis.	Journal Article; Video-Audio Media	eng	Surgical endoscopy	10.1007/s00464-020-07383-0	https://pubmed.ncbi.nlm.nih.gov/31953732/
31676330	Carmicheal J	2020	Elevating pancreatic cystic lesion stratification: Current and future pancreatic cancer biomarker(s).	Non-primary publication	Non-primary publication (review/editorial/letter/guideline)	Journal Article; Research Support, N.I.H., Extramural; Review	eng	Biochimica et biophysica acta. Reviews on cancer	10.1016/j.bbcan.2019.188318	https://pubmed.ncbi.nlm.nih.gov/31676330/
31385172	Merath K	2020	Use of Machine Learning for Prediction of Patient Risk of Postoperative Complications After Liver, Pancreatic, and Colorectal Surgery.	Wrong or non-separable population/procedure	Mixed liver/pancreatic/colorectal complication model without separable pancreas-specific performance in the report.	Journal Article	eng	Journal of gastrointestinal surgery : official journal of the Society for Surgery of the Alimentary Tract	10.1007/s11605-019-04338-2	https://pubmed.ncbi.nlm.nih.gov/31385172/
31826598	Zhang JL	2019	[Preoperative evaluation using multi-slice spiral CT angiography of right-side colon vascular in laparoscopic radical operation for right colon cancer].	Non-English publication	Non-English publication	Journal Article	chi	Zhonghua wai ke za zhi [Chinese journal of surgery]	10.3760/cma.j.issn.0529-5815.2019.12.011	https://pubmed.ncbi.nlm.nih.gov/31826598/
31510731	Qian ZY	2019	[Preliminary application of endoscopic titanium clip localization combined with three-dimensional CT reconstruction in the determination of resection margin of gastric central cancer under laparoscopy].	Non-English publication	Non-English publication	Journal Article	chi	Zhonghua wai ke za zhi [Chinese journal of surgery]	10.3760/cma.j.issn.0529-5815.2019.10.008	https://pubmed.ncbi.nlm.nih.gov/31510731/
31117111	Kuwahara T	2019	Usefulness of Deep Learning Analysis for the Diagnosis of Malignancy in Intraductal Papillary Mucinous Neoplasms of the Pancreas.	Task outside focused perioperative/technical resection scope	Diagnostic/characterisation study without direct pancreatic-resection application	Evaluation Study; Journal Article; Research Support, Non-U.S. Gov't	eng	Clinical and translational gastroenterology	10.14309/ctg.0000000000000045	https://pubmed.ncbi.nlm.nih.gov/31117111/
30929369	Fang CH	2019	[Construction and application of the core technology system of digital intelligent diagnostic and treatment for hepato-biliary-pancreatic diseases].	Non-English publication	Non-English publication	Journal Article	chi	Zhonghua wai ke za zhi [Chinese journal of surgery]	10.3760/cma.j.issn.0529-5815.2019.04.003	https://pubmed.ncbi.nlm.nih.gov/30929369/
30803366	Wei R	2019	Computer-Aided Diagnosis of Pancreas Serous Cystic Neoplasms: A Radiomics Method on Preoperative MDCT Images.	Task outside focused perioperative/technical resection scope	Diagnostic/characterisation study without direct pancreatic-resection application	Journal Article; Research Support, Non-U.S. Gov't	eng	Technology in cancer research & treatment	10.1177/1533033818824339	https://pubmed.ncbi.nlm.nih.gov/30803366/
29981604	Tomimaru Y	2018	The Branching Pattern of the Inferior Pancreaticoduodenal Artery in Patients with a Replaced Right Hepatic Artery.	No eligible AI/ML/computational method	Anatomical imaging study; no eligible AI/ML resection application.	Journal Article	eng	The American surgeon		https://pubmed.ncbi.nlm.nih.gov/29981604/
28478192	Menderes G	2018	Natural Orifice-Assisted Laparoscopic Meckel Diverticulectomy Incidentally Found During Para-Aortic Mass Resection.	Wrong or non-separable population/procedure	Wrong procedure/population.	Case Reports; Journal Article; Video-Audio Media	eng	Journal of minimally invasive gynecology	10.1016/j.jmig.2017.04.015	https://pubmed.ncbi.nlm.nih.gov/28478192/
28290711	Qu J	2017	Visualising a rare and complex case of advanced hilar cholangiocarcinoma.	Wrong or non-separable population/procedure	Cholangiocarcinoma visualization case; wrong disease/procedure.	Journal Article	eng	Journal of visual communication in medicine	10.1080/17453054.2017.1289060	https://pubmed.ncbi.nlm.nih.gov/28290711/
27261244	Fung A	2016	Creating an animation-enhanced video library of hepato-pancreato-biliary and transplantation surgical procedures.	No eligible AI/ML/computational method	Educational video-library development; no eligible AI/ML pancreatic-resection analysis.	Journal Article	eng	Journal of visual communication in medicine	10.1080/17453054.2016.1182474	https://pubmed.ncbi.nlm.nih.gov/27261244/
24444460	Smith BJ	2014	An interactive Bayesian model for prediction of lymph node ratio and survival in pancreatic cancer patients.	Task outside focused perioperative/technical resection scope	Bayesian model predicts lymph-node ratio and survival; oncological prognostication without direct perioperative resection application.	Journal Article; Research Support, N.I.H., Extramural	eng	Journal of the American Medical Informatics Association : JAMIA	10.1136/amiajnl-2013-002171	https://pubmed.ncbi.nlm.nih.gov/24444460/
24273270	Hou B	2013	[Application of three-dimensional visualization technology for laparoscopic resection of cystic carcinoma in the pancreatic body and tail].	Non-English publication	Non-English publication	Journal Article; Research Support, Non-U.S. Gov't	chi	Nan fang yi ke da xue xue bao = Journal of Southern Medical University		https://pubmed.ncbi.nlm.nih.gov/24273270/
23052528	Nissen NN	2013	Universal multifunctional HD video system for minimally invasive, [corrected] open and microsurgery.	Wrong or non-separable population/procedure	General video-system technology; no pancreas-specific AI/resection analysis.	Evaluation Study; Journal Article	eng	Surgical endoscopy	10.1007/s00464-012-2552-5	https://pubmed.ncbi.nlm.nih.gov/23052528/
22832999	Seeliger B	2012	Duodenal duplication cyst: a potentially malignant disease.	Wrong or non-separable population/procedure	Non-pancreatic disease; wrong population.	Case Reports; Journal Article	eng	Annals of surgical oncology	10.1245/s10434-012-2502-4	https://pubmed.ncbi.nlm.nih.gov/22832999/
22736284	Vemuri AS	2012	Deformable three-dimensional model architecture for interactive augmented reality in minimally invasive surgery.	Wrong or non-separable population/procedure	Generic augmented-reality model architecture; no pancreas-specific resection cohort/application.	Journal Article	eng	Surgical endoscopy	10.1007/s00464-012-2395-0	https://pubmed.ncbi.nlm.nih.gov/22736284/
22568419	Rebibo L	2012	Three-dimensional computed tomography analysis of the left gastric vein in a pancreatectomy.	No eligible AI/ML/computational method	Conventional 3D CT anatomy study; no AI/ML component.	Journal Article	eng	HPB : the official journal of the International Hepato Pancreato Biliary Association	10.1111/j.1477-2574.2012.00468.x	https://pubmed.ncbi.nlm.nih.gov/22568419/
21713410	Yu HC	2012	Computer-assisted three-dimensional reconstruction of the fetal pancreas including the supplying arteries according to immunohistochemistry of pancreatic polypeptide.	Wrong or non-separable population/procedure	Fetal/anatomical reconstruction; not human pancreatic resection.	Journal Article; Research Support, Non-U.S. Gov't	eng	Surgical and radiologic anatomy : SRA	10.1007/s00276-011-0844-4	https://pubmed.ncbi.nlm.nih.gov/21713410/
20037847	Maruyama Y	2010	Perioperative challenges associated with a pancreaticoduodenectomy and distal pancreatectomy for pancreatic cancer in patients with situs inversus totalis: report of two cases.	No eligible AI/ML/computational method	Pancreatic surgery case reports; no eligible AI/ML method.	Case Reports; Journal Article	eng	Surgery today	10.1007/s00595-009-4064-z	https://pubmed.ncbi.nlm.nih.gov/20037847/

PMID	Study	Year	Title	Detailed-assessment exclusion category	Specific exclusion reason	Publication type	Language	Journal	DOI	PubMed URL
18712270	Baque P	2009	Division of the right posterior attachments of the head of the pancreas with a linear stapler during pancreaticoduodenectomy: vascular and oncological considerations based on an anatomical cadaver-based study.	Wrong or non-separable population/procedure	Cadaveric anatomical study; not an eligible human resection AI study.	Journal Article	eng	Surgical and radiologic anatomy : SRA	10.1007/s00276-008-0353-2	https://pubmed.ncbi.nlm.nih.gov/18712270/
17191428	Maeda H	2006	Usefulness of multi-detector row computed tomography for accurate preoperative assessment of pancreatic adenocarcinoma: report of a case.	No eligible AI/ML/computational method	Diagnostic CT case report; no eligible AI/ML resection application.	Case Reports; Journal Article	eng	West African journal of medicine	10.4314/wajm.v25i3.28287	https://pubmed.ncbi.nlm.nih.gov/17191428/
41836620	Yoon J	2026	Machine learning-based prediction of long-term new-onset diabetes mellitus risk after pancreaticoduodenectomy using radiomics.	Task outside focused perioperative/technical resection scope	Long-term metabolic sequela prediction (new-onset diabetes/NAFLD) was outside the focused perioperative and technical surgical scope.	Journal Article	eng	Digital health	10.1177/20552076261415943	https://pubmed.ncbi.nlm.nih.gov/41836620/
40395025	Wang Y	2025	Evaluation of tumor pseudocapsule using computed tomography-based radiomics in pancreatic neuroendocrine tumors to predict prognosis and guide surgical strategy: a cohort study.	Task outside focused perioperative/technical resection scope	Oncological staging, prognosis, or surgical-selection endpoint without a short-term perioperative or technical resection endpoint; excluded to preserve the focused scope.	Journal Article	eng	International journal of surgery (London, England)	10.1097/JS9.0000000000002511	https://pubmed.ncbi.nlm.nih.gov/40395025/
40288090	Ahmed KS	2025	Machine Learning to Predict Early Death Despite Pancreaticoduodenectomy.	Task outside focused perioperative/technical resection scope	Oncological staging, prognosis, or surgical-selection endpoint without a short-term perioperative or technical resection endpoint; excluded to preserve the focused scope.	Journal Article; Research Support, N.I.H., Extramural	eng	The Journal of surgical research	10.1016/j.jss.2025.03.047	https://pubmed.ncbi.nlm.nih.gov/40288090/
38985503	Janssen BV	2024	Artificial Intelligence-based Segmentation of Residual Pancreatic Cancer in Resection Specimens Following Neoadjuvant Treatment (ISGPP-2): International Improvement and Validation Study.	Task outside focused perioperative/technical resection scope	AI analysis of treatment response in resection specimens without a direct perioperative, margin, or operative-guidance endpoint; outside the focused scope.	Journal Article; Multicenter Study; Validation Study	eng	The American journal of surgical pathology	10.1097/PAS.0000000000002270	https://pubmed.ncbi.nlm.nih.gov/38985503/
38696129	Mapelli P	2024	Preoperative assessment of lymph nodal metastases with [(68)Ga]Ga-DOTATOC PET radiomics for improved surgical planning in well-differentiated pancreatic neuroendocrine tumours.	Task outside focused perioperative/technical resection scope	Oncological staging, prognosis, or surgical-selection endpoint without a short-term perioperative or technical resection endpoint; excluded to preserve the focused scope.	Journal Article	eng	European journal of nuclear medicine and molecular imaging	10.1007/s00259-024-06730-w	https://pubmed.ncbi.nlm.nih.gov/38696129/
38581555	Fujii T	2024	Radiomics-based prediction of nonalcoholic fatty liver disease following pancreatoduodenectomy.	Task outside focused perioperative/technical resection scope	Long-term metabolic sequela prediction (new-onset diabetes/NAFLD) was outside the focused perioperative and technical surgical scope.	Journal Article	eng	Surgery today	10.1007/s00595-024-02822-0	https://pubmed.ncbi.nlm.nih.gov/38581555/
38106556	Gu W	2023	Development and validation of CT-based radiomics deep learning signatures to predict lymph node metastasis in non-functional pancreatic neuroendocrine tumors: a multicohort study.	Task outside focused perioperative/technical resection scope	Oncological staging, prognosis, or surgical-selection endpoint without a short-term perioperative or technical resection endpoint; excluded to preserve the focused scope.	Journal Article	eng	EClinicalMedicine	10.1016/j.eclinm.2023.102269	https://pubmed.ncbi.nlm.nih.gov/38106556/
37018307	Hu P	2023	Prediction of New-Onset Diabetes After Pancreatectomy With Subspace Clustering Based Multi-View Feature Selection.	Task outside focused perioperative/technical resection scope	Long-term metabolic sequela prediction (new-onset diabetes/NAFLD) was outside the focused perioperative and technical surgical scope.	Journal Article	eng	IEEE journal of biomedical and health informatics	10.1109/JBHI.2022.3233402	https://pubmed.ncbi.nlm.nih.gov/37018307/

Supplementary Figure S1. Study-selection flow

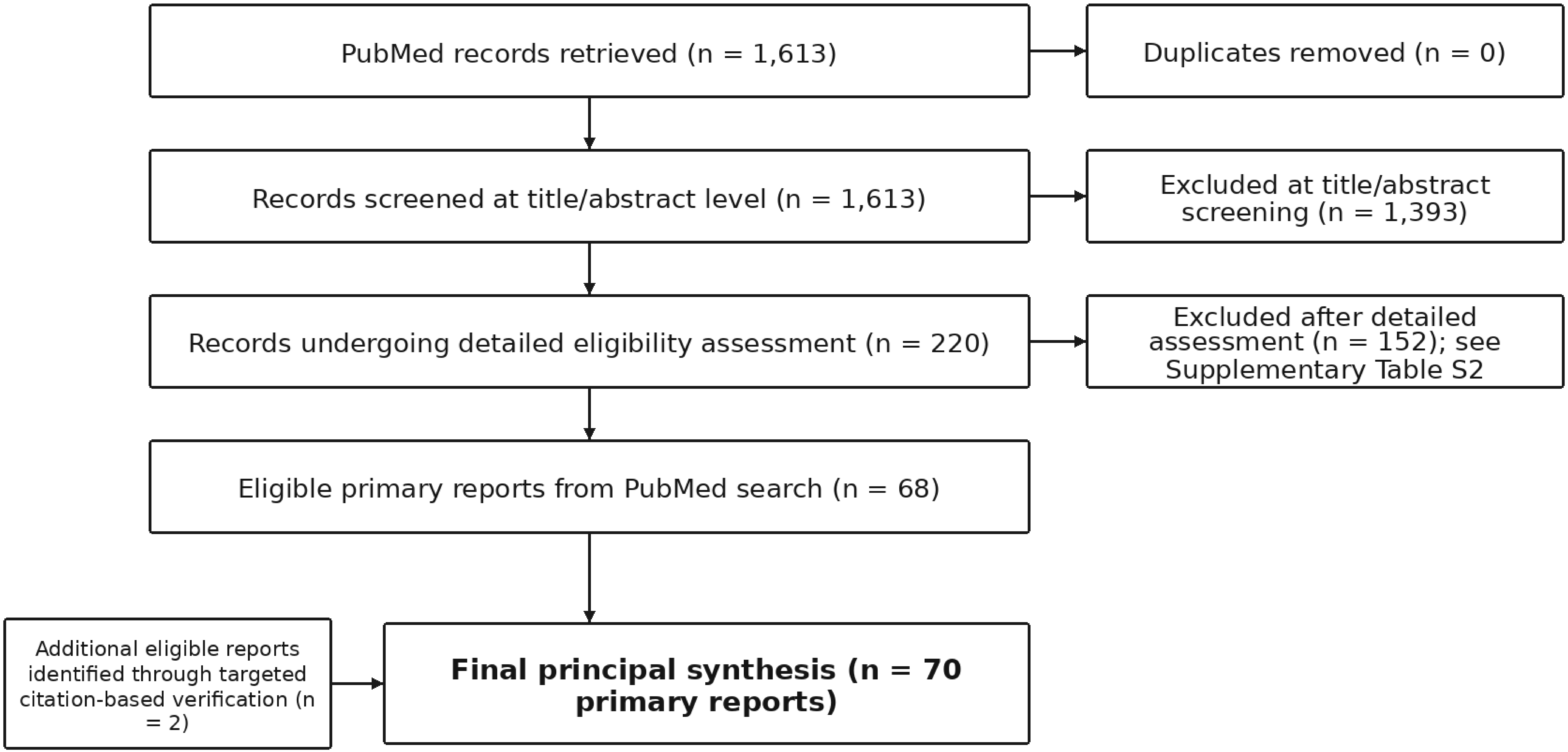

Evidence cutoff: 24 July 2026

No language, article-type, or human-species filter was applied at the search stage; English language was applied during eligibility screening.
